# Depopulation and aging–challenges for Croatia's climate resilience

**DOI:** 10.3389/fsoc.2025.1564299

**Published:** 2025-06-18

**Authors:** Ana-Maria Boromisa

**Affiliations:** Institute for Development and International Relations, Zagreb, Croatia

**Keywords:** climate change, adaptation, mitigation, resilience, aging, jobs, Croatia, depopulation

## Abstract

**Introduction:**

Climate change and the implementation of mitigation and adaptation policies have significant socioeconomic implications. Conversely, socioeconomic developments shape the capacity to design and enforce climate policies, creating a feedback loop. This paper localizes the impact of Climate change in Croatia and explores the feedback between socio-economic development and climate change. The starting hypothesis is that limitations in human capital critically hinder climate-resilient development in Croatia.

**Methods:**

The study uses climate data and literature to contextualize Croatia's climate vulnerability. A sector-specific analysis is conducted to identify key sectors for climate-resilient development based on their potential for emissions reduction, climate vulnerability, and current and potential economic importance. The current economic importance is evaluated based on contribution to GDP and employment, and key opportunities and obstacle for future growth are identified.

**Results:**

Climate data and literature indicate that Croatia, a high-income EU member, is experiencing warming faster than the global average and ranks among the least climate-resilient high-income countries. A sector-specific analysis identifies the most critical sectors for Croatian climate-resilient development based on their emission reduction potential, climate vulnerability, and growth opportunities. Among these are sectors that contribute the most to GDP and employment—such as tourism, construction, and healthcare —which already suffer from significant labor shortages. The results indicate that a shrinking workforce is the key constraint for implementation of climate-resilient development.

**Discussion:**

Significant improvements in labor productivity, higher participation rates, integration of foreign workers in the labor market, and efforts to address skills shortages are necessary. This presents a challenge, given increasing damages from extreme climate events, ongoing depopulation, a limited supply of a highly educated workforce, and low participation in lifelong learning. For climate-resilient development, it is essential to design policies that adequately address aging and depopulation –both of which limit economic growth and reduce capacity for climate adaptation.

**Conclusions:**

Findings of the Croatian case offer insights for Mediterranean and island countries reliant on climate-sensitive sectors like tourism (e.g., Greece, Thailand). The high-income yet low-resilience paradox is relevant for regions such as Southern Europe, Australia, and California. EU membership highlights institutional misalignments between supranational climate agendas (e.g., the European Green Deal) and subnational demographic realities. These dynamics are relevant to aging societies (Japan, Germany) and post-industrial economies (Poland, Canada) navigating green transitions, emphasizing the need to integrate demographic strategies into climate governance.

## 1 Introduction

The effects of the climate crisis are expected to become increasingly pronounced in the coming years, posing an existential threat (IPCC, 2021, 2022a; World Economic Forum, 2021).

A growing body of literature estimates the losses and damage resulting from climate change (IPCC, 2022a; Mastrorillo et al., 2016; Stabroek and Jansen, 2021; Schwerdtle et al., 2018; UNHRC, 2024; Adom, 2024). In general, the costs of inaction exceed those associated with proactive measures (Stern, 2006; Metcalf and Weisbach, 2009; Dietz et al., 2018; IPCC, 2014; Dasgupta, 2008, 2021).

The transition toward climate neutrality mitigates climate change, while the implementation of adaptation policies enables societies to cope with its unavoidable effects. Climate change and climate policies drive socio-economic transformation, though the impacts of this transformation vary across communities, regions, and economic sectors. Developed countries are more resilient to climate change due to their stronger economic structures, advanced technologies, and well-established governance systems. However, vulnerable areas—including islands, remote territories, rural communities, mountainous regions, and less developed areas—face significant challenges that exacerbate inequalities.

Croatia's GDP per capita is 45% of the EU average at market prices and 76% of the EU average when measured in purchasing power parity (data for 2024, Eurostat, 2025a,b). Only Bulgaria and Romania have lower values in both indices. Based on GDP per capita, Croatia ranks among the least economically developed EU countries.

Among the country's four regions, GDP per capita varies significantly relative to the national average. The most developed region, the City of Zagreb, has a GDP per capita at 176% of the national average, whereas the least developed region, Pannonian Croatia, stands at just 66% of the national average (DZS, 2025).

EU-wide studies indicate that the transition to a low-carbon economy is likely to result in a net increase in employment (Asikainen et al., 2021; European Commission, 2020; CEDEFOP, 2021; Eurofound, 2023; Vandeplas et al., 2022; Murauskaite-Bull et al., 2021). However, Croatia is already facing a shortage of workers in several sectors, including healthcare, tourism, and construction. The number of jobs created during the transition process will depend on demand and investment in greener products and services. Moreover, policies will influence the demand for workers in specific sectors, the pace of the transition, the coherence between the green and digital transitions, and the skills required for new jobs.

Existing research highlights the importance of demographic factors in shaping both climate resilience and the broader socio-economic impacts of climate policies. There is a mismatch between population growth rates and emission levels across countries, as well as a complex relationship between demographic changes and societies' adaptive capacity to cope with climate change (Deuster et al., 2023; Muttarak, 2021; Chapagain et al., 2025). Population aging is identified as a key factor driving future heat- and cold-related mortality, with increasing deaths projected in aging societies (Chen et al., 2024b).

However, a significant gap remains in understanding the feedback mechanisms involved—specifically, how socio-economic developments influence the ability to design and implement climate policies. There is a notable scarcity of studies examining the socio-economic impacts of climate policies specifically in Croatia. This paper seeks to address this knowledge gap. It applies global knowledge on the socio-economic impacts of climate policies to the Croatian context, assesses the country's current level of climate resilience, and explores strategies to enhance resilience during the transition to climate neutrality.

The starting hypothesis is as follows: implementation of climate policies in Croatia is hindered by limitations in human capacity. Climate polices do not sufficiently consider aging and depopulation which limits growth potential and decrease ability to climate resilient development.

The literature identifies various climate resilience discourses, such as neoliberal, reactive, security-based resilience, ecological and transformational (Ferguson et al., 2021). Resilience can be viewed as the inverse of vulnerability (Walker et al., 2002). IPCC's definitions of resilience show that this is an evolving concept. Its 2012 report “Managing the Risks of Extreme Events and Disasters to Advance Climate Change Adaptation” defines resilience as “the ability of a system to anticipate, absorb, accommodate or recover from the effects of a hazardous event” (Field et al., 2012). The Sixth Assessment Report defines resilience as “capacity of interconnected social, economic and ecological systems to cope with a hazardous event, trend or disturbance, responding or reorganizing in ways that maintain their essential function, identity and structure. Resilience is a positive attribute when it maintains capacity for adaptation, learning and/or transformation” (Carson and Peterson, 2016; IPCC, 2022a). The updated definition considers both acute threats, identified as “event and disturbance” and chronic changes, identified as “trend” caused by climate change (Table 1). The resilience related taxonomies, such as EU taxonomy (European Commission, 2021a), UNDP SDG Finance Taxonomy (UNDP, 2020) or Sendai Framework for Disaster Risk Reduction 2015–2030 (United Nations Office for Disaster Risk Reduction, 2015) also consider acute and chronic hazards.

**Table 1:** Categorization of chronic and acute hazards.

**Type of hazard**	**Temperature**	**Wind**	**Water**	**Solid mass**
Chronic hazards	Change (air, fresh water, sea water) Temperature stress Temperature variability	Change in wind currents	Changes in precipitation patterns and types (rain, hail, snow, ice) Precipitation variability or hydrological variability Acidification of the sea Salt water penetration Sea level rise Water shortage	Coastal erosion Soil erosion Soil degradation Solifluction
Acute hazards	Heat wave Cold wave Frost Forest fire	Storm (blizzard, stormy wind with dust) Leech	Drought Intense precipitation (rain, hail, ice, snow) Floods (precipitation, river, coastal)	Slide Soil subsidence

Source: European Commission (2021b).

This paper adopts the IPCC's definition of resilience as “the capacity of interconnected social, economic, and ecological systems to cope with a hazardous event, trend, or disturbance by responding or reorganizing in ways that maintain their essential function, identity, and structure.” Additionally, climate-resilient development is defined as the process of implementing greenhouse gas mitigation and adaptation measures to support sustainable development for all (IPCC, 2022a). The Notre Dame -Global Adaptation Initiative (ND-GAIN) country index (University of Notre Dame, 2025) and EU taxonomy methodology for evaluation vulnerability (as inverse of resilience; European Commission, 2021b), are used to assess sector-specific needs and opportunities for improving climate resilience.

The methodology section that follows (section 2) provides information on methodological approach and conceptual framework, data sources, projections and scenarios used. Presentation of results (section 3) follows methodological approach used. Section 3.1. presents climate data and observed changes for Croatia, together with identified trends and scenarios. Section 3.2. integrates knowledge on impacts of climate change on socio-economic transformation, based on systematic literature review. Section 3.3. provides Croatian context in terms of demography and labor market trends, section 3.4. presents GDP scenarios and section 3.5. provides an overview of sector-specific vulnerabilities and opportunities.

Section 4 discusses the results, highlighting key challenges to climate-resilient development (CRD) in Croatia. It presents the current level of climate resilience, assesses Croatia's capacity to enhance it and implement EU climate policies, and identifies aging and depopulation as the main barriers to policy implementation. Additionally, it analyses the feedback loop, focusing on the impact of demographic trends—aging and depopulation—on CRD.

Conclusions (section 5) summarize how the findings address knowledge gaps on climate change impacts and the feedback effects of socio-economic developments on climate policy capacity. The section also offers perspective for future work and indicates relevance of Croatian case study for other countries.

## 2 Methodology

Established research on climate change and its socio-economic impacts serves as the foundation for contextualizing these findings in Croatia.

**Figure 1:**
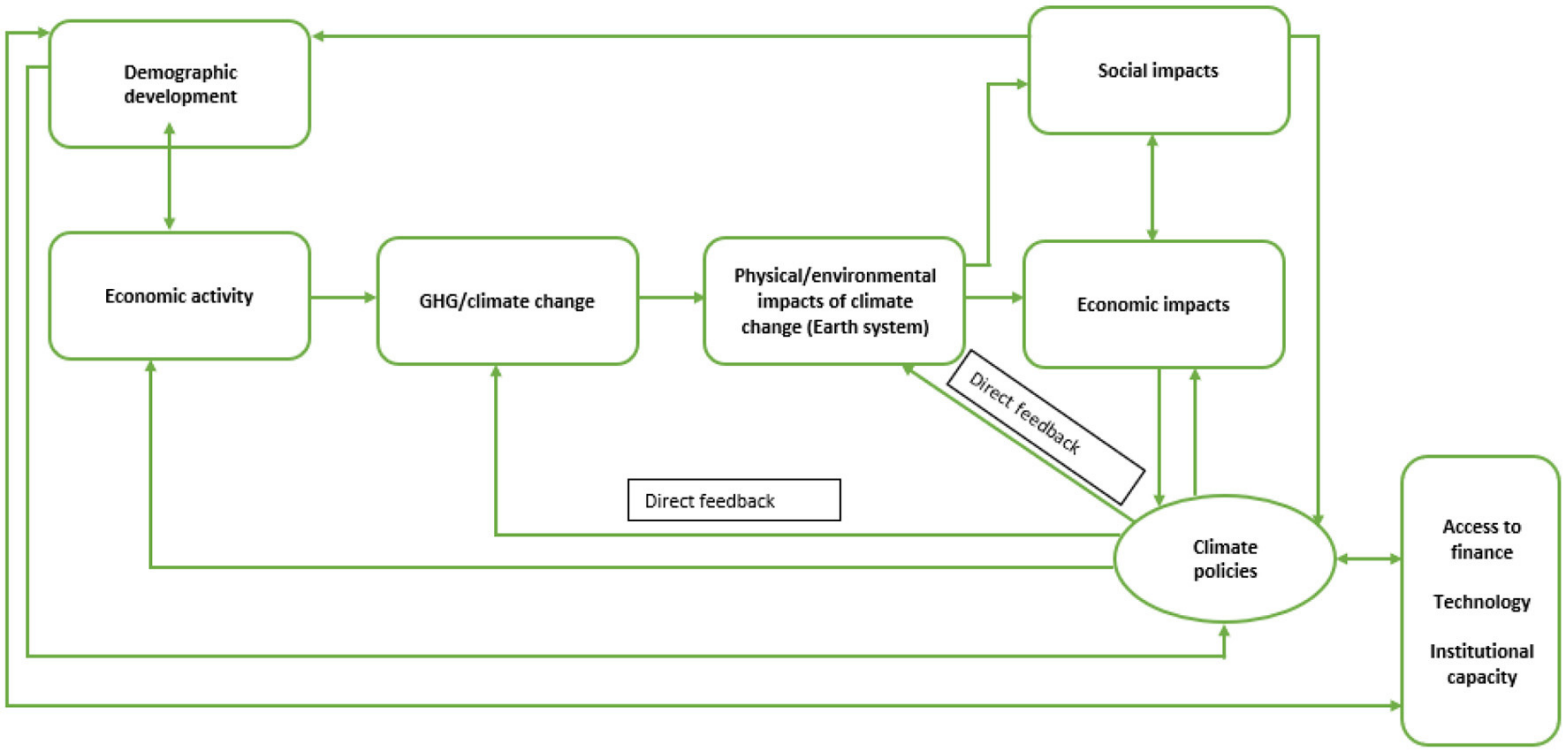
Global interlinkages between economic activity, climate change and society. Source: author.

**Figure 2:**
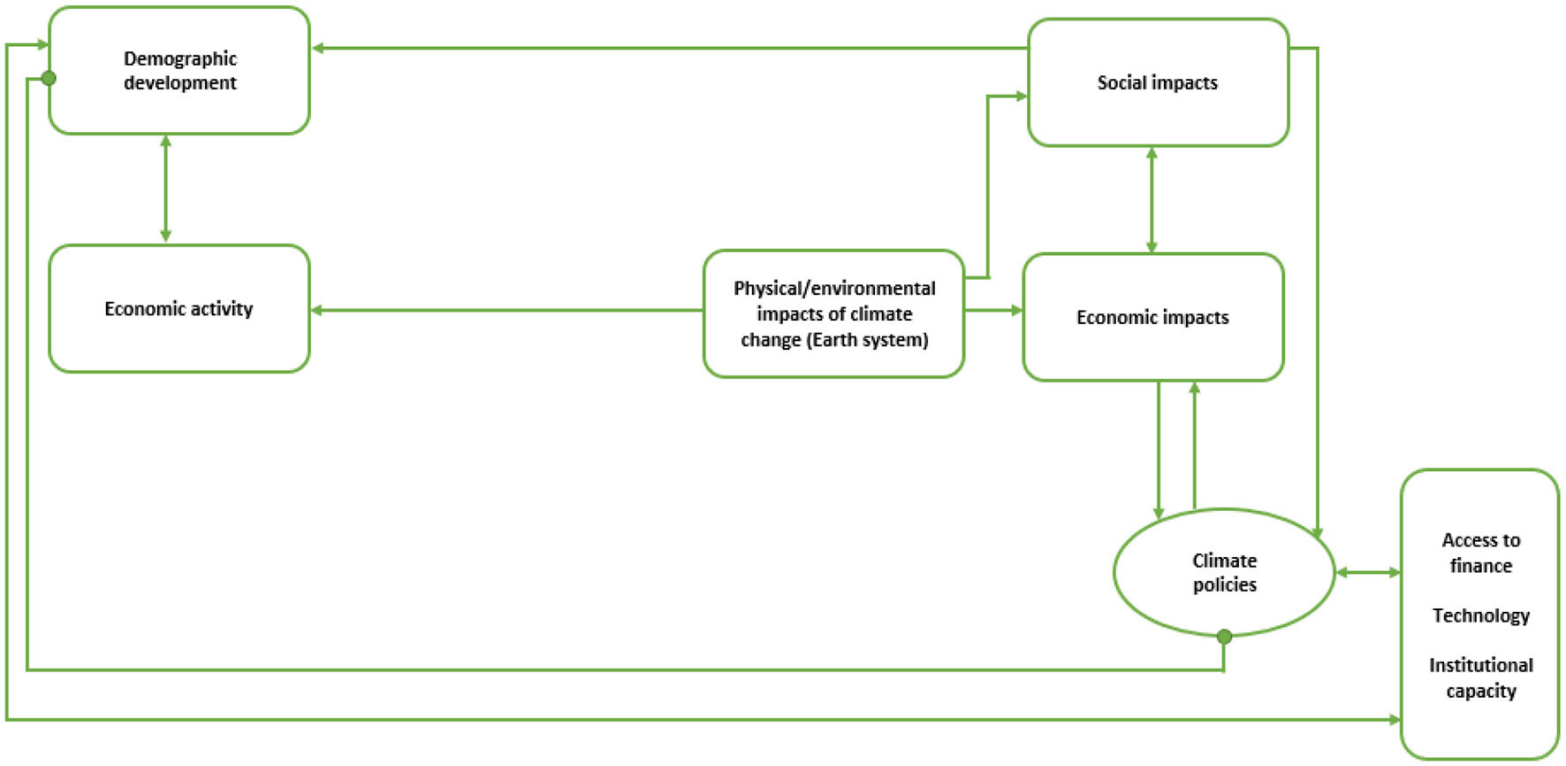
Interlinkages between economic activity, climate change and society at national level (small state). Source: author.

The conceptual framework for this analysis builds on existing knowledge of climate change, its sources and impacts, together with role of climate policies and their dependency on access to finance, technology and institutional capacity, as illustrated in Figure 1. There is evidence of direct impact of global GHG emissions on climate change which causes economic and social consequences. This global-level model reflects the dynamics observed in major emitting countries, where economic activity leads to substantial GHG emissions, triggering climate feedback loops that, in turn, generate socio-economic consequences. Feedback mechanism is direct. The link between anthropogenic GHG emissions and climate change is not evident in small economies. In these contexts, localization of interlinkages between economic activity and climate change disrupts the straightforward connection observed at the global level, as illustrated in Figure 2. Consequently, in small countries the processes and feedbacks operate through different channels. The framework thus emphasizes the indirect transmission of global climate impacts through economic dependencies, demographic structures, and governance systems, highlighting a different logic of vulnerability and resilience.

Five step integrative methodology was applied to assess the implications of climate change for Croatia's socio-economic development, resilience, and labor market transformation. The methodological design reflects the interdisciplinary nature of the research, combining climate science, demographic analysis, economic projections, and sectoral assessments. Each step includes data collection, literature review (including peer reviewed literature and gray reports), integration and systematization of existing knowledge and serves as input for following steps. To ensure data accuracy and reliability, and enable replicability, internationally harmonized assumptions and projections are used, while national data was used for localization. Table 2 summarizes and structures data sources used in each step, across the domains of climate change and economic/demographic data.

**Table 2:** Data sources used.

**Step no**	**National statistics**	**Government ministries and strategy documents**	**International organizations**	**Scenarios, reference, impact assessment**
1 *Climate trends and scenarios*	**Climate**: DHMZ (temperature, precipitation, extremes) **Economic/Demographic**: DZS census baseline for regional analysis	National adaptation strategy (Croatian Parliament, 2020)	World Bank Climate Knowledge Portal; UNEP; IMF; WMO; Eurostat	AR6 climate scenarios (SSP1–1.9 to SSP5–8.5); exposure analysis by region
2 *Socio-economic impacts*	**Climate**: DZS mortality data (heatwaves) **Economic/demographic**: DZS GDP, labor force	National energy and climate plan (NECP); health development plan	ILO, Eurofound, CEDEFOP, WEF, EEA	ILO projections (labor losses from heat); EU Green Deal policy scenarios
3 *Demographic trends and labor supply*	**Economic/demographic:** 2021 census; labor force survey; employment by sector	N/A (Projections not updated nationally)	UN World Population Prospects, Eurostat, World Bank Population Projections	UN, Eurostat, and World Bank population projections under baseline and sensitivity variants
4 *Economic projections and productivity*	**Economic/demographic:** DZS: GDP (total and per capita), employment, wages	National development strategy	Eurostat (EU GDP benchmarks), World Bank	EU convergence targets; productivity benchmarks tied to population scenarios (from Step 3)
5 *Sectoral assessment*	**Economic/demographic:** DZS: sectoral GDP/employment; wage data; ministry of the interior (work permits)	NECP; low-carbon strategy; tourism and health strategies; MINGOR reports	IRENA, ILO, Transparency International, Eurostat	SSP2–4.5 and SSP3–7.0 used for qualitative vulnerability matrix; climate hazard linkage to sectoral systems

Source: author.

### 2.1 Step 1: climate change trends and scenario framework

This step synthesizes key findings from the Intergovernmental Panel on Climate Change (IPCC) Sixth Assessment Report (AR6) to establish a baseline understanding of global and regional climate change trajectories (IPCC, 2022b). To support interpretation of localized projections, particular attention is given to the Shared Socioeconomic Pathways (SSPs) and Representative Concentration Pathways (RCPs) frameworks used in AR6. These are summarized in Tables 3, 4, respectively, and further combined into five scenario pairings (SSP1-1.9, SSP1-2.6, SSP2-4.5, SSP3-7.0, and SSP5-8.5; Table 5). These integrated SSP-RCP combinations are designed to capture a comprehensive range of future climate conditions, shaped by varying socio-economic trends and emissions levels. Their explanation provides a conceptual foundation for understanding the assumptions and implications of country-specific projections used later in the analysis.

**Table 3:** Shared socioeconomic pathways (SSPs).

**SSP dimension**	**SSP1 sustainability-taking the green road**	**SSP2 middle of the road**	**SSP3 regional rivalry-a rocky road**	**SSP4 inequality-a road divided**	**SSP5 fossil-fueled development-taking the highway**
Population (2100)	~7 billion (peak and decline)	~9 billion	~13 billion	~9 billion	~7 billion (peak and decline)
Income	High	Medium	Low	Medium	High
Inequality	Reduced	Gradual reduction	Continued	Significant within and across regions	Reduced
Technological progress	High	Medium	Low	Medium	High
Consumption and production	Less resource-intensive, low-GHG food, less waste	Continuation of past trends	Material-intensive	Varied	Resource-intensive
Trade	Free trade	Moderate	Trade barriers	Mixed	Free trade
Land use and environment	Effective regulation, eco-friendly	Moderate regulation	Poor regulation	Mixed effectiveness	Tech-focused, less sustainable
Challenges to mitigation	Low	Medium	High	Low	High
Challenges to adaptation	Low (High adaptive capacity)	Medium (Medium adaptive capacity)	High (Low adaptive capacity)	High (Low adaptive capacity)	Low (High adaptive capacity)

Source: Author, based on Riahi et al. (2017).

**Table 4:** RCPs.

**RCP parameter**	**RCP 2.6 low emission scenario**	**RCP 4.5 intermediate emissions scenario**	**RCP 6.0 intermediate high emission scenarios**	**RCP 8.5 high emission scenario, “business-as usual”**
Radiative Forcing Peak	~3 W/m^2^ mid-century	Stabilization	Stabilization	Rising
Radiative Forcing in 2,100	2.6 W/m^2^	4.5 W/m^2^	6.0 W/m^2^	8.5 W/m^2^
Trajectory description	Peak and decline; achieving net-zero CO_2_ emissions before 2,100	Stabilization without overshoot (post-2,100)	Stabilization without overshoot (post-2,100)	Continuous rise to 2,100
Mitigation efforts	Immediate and ageressive	Moderate, emissions peaking around mid-century	Delayed	Limited, business as usual
Peak warming	1.5°C−2°C above pre-industrial levels	~2.5°C−3°C by 2,100	reaches ~3.5°C−4°C by 2,100	exceeding ~4°C−5°C by 2,100.

Source: author, based on RCP database 2.0 (2025).

**Table 5:** Link between SSPs and RCPs.

**Scenario**	**Emissions level**	**Description**	**Projected warming by 2,100**
**SSP1-1.9**	Very Low	Sustainability-focused world with strong global cooperation and rapid decarbonization. Net zero CO_2_ emissions achieved around 2050	~1.5°C
**SSP1-2.6**	Low	Similar to SSP1-1.9 but with slower emission reductions.	~2°C
**SSP2-4.5**	Intermediate	“Middle of the Road” scenario reflecting current policies with no major mitigation shifts.	~2.7–3°C
**SSP3-7.0**	High	“Regional Rivalry” scenario with weak climate policies, slow tech progress, and fossil fuel reliance.	~3.5–4°C
**SSP5-8.5**	Very High	“Fossil-Fuel Driven” scenario with high economic growth and intensive fossil fuel use.	>4°C

Source: author.

Building on this framework, climate impacts in Croatia were assessed using downscaled projections from the World Bank's Climate Knowledge Portal, which applies the AR6 scenario structure. These projections capture localized changes in temperature, precipitation, extreme events, and regional climate anomalies. Emphasis is placed on interpreting these trends within the SSP2-4.5 and SSP3-7.0 scenarios, which reflect current global policy trends and regionally differentiated risks. The localized data provide the empirical basis for the sectoral and socio-economic analyses that follow in later steps.

### 2.2 Step 2: socio-economic impacts and labor market vulnerability

This step provides an overview of the impacts of climate change on socio-economic development, drawing on global and EU-level evidence. It synthesizes available findings on how climate change affects macroeconomic performance, social inequality, employment, and productivity. A literature-based impact assessment approach was applied, combining quantitative estimates (e.g., GDP loss per degree of warming) with qualitative insights into inequality dynamics and sector-specific labor disruptions. At the national level, overall resilience was assessed using the ND-GAIN index (Gain.nd.edu., 2024), while sectoral vulnerabilities were identified through a screening process based on indicators of sensitivity and exposure (European Commission, 2021a).

### 2.3 Step 3: demographic scenarios and labor supply projections

Step three models labor market development using a demographic-economic scenario approach, which integrates population projections with employment modeling. Employment estimates are aligned with projected declines in the working-age population. To determine the requirements for stabilizing employment levels in the context of a shrinking working-age population, the necessary increase in labor force participation was estimated. This was achieved by applying population projections disaggregated by age and sex to calculate the number of additional individuals—particularly women and older adults—who would need to enter or remain in the labor market under each scenario. The analysis accounted for fixed or variable unemployment and activity rates, depending on the assumptions of the respective scenarios.

Two scenarios were developed: (i) *Scenario 1* assumes a constant number of active persons and a fixed unemployment rate at 2021 levels—that is, an active population of 1.816 million and an unemployment rate of 7.5%; (ii) *Scenario 2* assumes a constant activity rate of 73% (as recorded in 2021), with the unemployment rate varying within a range of 5–10%.

### 2.4 Step 4: economic projections and productivity requirements

This step develops two GDP scenarios for Croatia using a macroeconomic modeling approach aligned with the methodology applied to employment projections. In *Scenario 1*, calculations were performed to estimate the increase in GDP per capita required to stabilize total GDP in the context of a declining population. *Scenario 2* assumes convergence with long-term development goals and EU cohesion policy objectives, targeting 66% of the EU average GDP per capita by 2030 and full convergence (100%) by 2050.

Both scenarios were evaluated against the demographic projections outlined in Step 3. Required labor productivity improvements were estimated using the standard formula:


$$\begin{array}{l} Labour\;Productivity\;=\;\frac{Total\;GDP}{Number\;of\;Employed\;Persons} \end{array}$$

Projected labor force contraction was used to calculate the productivity growth thresholds necessary to maintain or increase GDP. Additionally, the analysis examined regional disparities in GDP per capita, with a particular focus on urban–rural and intra-regional inequalities.

### 2.5 Step 5: sectoral vulnerability and opportunity assessment

Step five assesses Croatia's economic structure and its interactions with climate change and related policies through an in-depth examination of five priority sectors. A multi-criteria assessment matrix was applied to evaluate and prioritize sectors across five dimensions: (i) contribution to GDP; (ii) contribution to employment; (iii) potential for reducing greenhouse gas (GHG) emissions; (iv) vulnerability to climate change; and (v) potential for growth driven by climate policies.

To ensure analytical consistency, the assessment integrated two complementary sectoral frameworks:

(i) the national economic structure, based on sectoral GDP and employment data from national statistics; and

(ii) the sectoral classifications used in Croatia's national climate policy documents, including the Low-Carbon Development Strategy, National Energy and Climate Plan (NECP), and Adaptation Strategy.

Five sectors identified as high-priority in both frameworks were selected for detailed analysis. These sectors were evaluated using scenario-based projections under SSP2-4.5 and SSP3-7.0. Out of the five SSP-RCP combinations introduced in Step 1, these two were further elaborated because the AR6 identifies them as the most plausible for medium-term planning, reflecting prevailing global emissions trajectories and socio-political trends.

The analysis also explores feedback loops between climate change, climate policies, and socio-economic development, illustrating how sectoral dynamics influence national resilience and transition pathways. The applied methodology enables the identification of key sources of vulnerability, current levels of resilience, and the main challenges to achieving climate-resilient development (CRD) across three time horizons: short term (to 2027), medium term (to 2030), and long term (to 2050). Special attention is given to the role of demographic trends—particularly aging and depopulation—as structural factors shaping the feasibility and direction of CRD.

## 3 Results

### 3.1 Climate data, trends and scenarios

The Intergovernmental Panel on Climate Change (IPCC) Sixth Assessment Report (AR6) provides comprehensive and up-to-date synthesis of climate science (IPCC, 2022b). It asserts that the average temperature of the Earth has increased by 1.09°C between 2011 and 2020, above the levels observed in 1850–1900, with increasingly severe and widespread impacts on ecosystems, human health, and economies (IPCC, 2022b).

According to the *IPCC Sixth Assessment Report* (IPCC, 2021), there is approximately a 50% likelihood that global warming will reach or exceed 1.5°C in the near term, even under a very low greenhouse gas emissions scenario. Furthermore, extreme weather events that historically occurred once every 10 years during the pre-industrial period (1850–1900) are now likely to occur 2.8 times every 10 years. If global warming reaches 1.5°C, this frequency is projected to increase to 4.1 times per decade. These climate-induced extremes are expected to intensify the risk of severe floods and droughts (IPCC, 2021).

AR6 reaffirms that human influence is unequivocally the dominant driver of global warming, with greenhouse gas (GHG) emissions from fossil fuel combustion, land-use changes, and industrial processes being the primary contributors (IPCC, 2021). AR6 provides five future scenarios, incorporating both socio-economic developments and emission pathways (SSPs and RCPs). Riahi et al. (2017) laid the groundwork for the SSPs, which became a core component of IPCC AR6 scenario analysis. SSPs are summarized in Table 2.

Representative Concentration Pathways (RCPs) are greenhouse gas concentration trajectories used in climate modeling to project future climate conditions. They represent different possible futures based on varying levels of greenhouse gas (GHG) emissions and radiative forcing. Four RCPs were first introduced in the IPCC Fifth Assessment Report (AR5) and named according to their radiative forcing levels (measured in watts per square meter, W/m^2^) by the year 2100. They are presented in Table 3.

The AR6 uses SSP-RCP combinations to create five updated climate scenarios (Table 4).

AR6 identifies SSP1-2.6 as the best-case scenario and SSP3-7.0 as the worst-case scenario, illustrating a future without new climate policies, where global temperatures by 2100 could rise between 3.1°C and 5.1°C above pre-industrial levels. SSP2-4.5 is an intermediate pathway, assumes that global socioeconomic trends largely follow historical patterns, with moderate efforts to reduce greenhouse gas emissions.

AR6 underscores regional disparities in climate impacts, with low-income and coastal regions being particularly vulnerable. In addition to mitigation efforts, AR6 stresses the urgency of adaptation measures, as some climate change effects are unavoidable. The findings of AR6 serve as the scientific foundation for international climate policy, reinforcing the need for immediate and coordinated global action to mitigate and adapt to the evolving climate crisis.

In Croatia, climate has changed over recent decades. Changes are characterized by (i) increased average temperatures (including higher surface temperatures of land and sea, and a greater number of consecutive hot and dry days), (ii) reduced precipitation, which is expected to intensify over time, along with more frequent and severe droughts, and (iii) increased frequency and intensity of extreme weather events (World Bank, 2024a). As a Mediterranean country, Croatia is warming 20% faster than the global average (UNEP, 2021; IMF, 2023). Precipitation patterns show significant regional variation. The Adriatic coast is experiencing a decline in annual precipitation, whereas other parts of the country exhibit mixed trends. Coastal zones are increasingly exposed to disaster risks, including flooding and erosion. Extreme weather events—such as cold and heat waves, floods, droughts, wildfires, and storms—are occurring with greater frequency.

The World Bank's Climate Knowledge Portal offers climate projections based on different models, using assumptions in accordance with the SSP1-1.9, SSP1-2.6, SSP2-4.5, SSP3-7.0, and SSP5-8.5 scenarios. Projections for Croatia suggest continued warming and further shifts in precipitation patterns. During summer (in June, July and August), precipitation is projected to decrease, while the number of hot days (with temperature exceeding 30°C) is projected to increase. Mean temperatures will rise, alongside an increase in the frequency and intensity of extreme weather events such as heatwaves and dry spells. Anomalies of average mean surface air temperature in Croatia have already reached 1°C compared to pre-industrial level (estimated at 11.4 C). Projections show that the anomaly has reached 1.5°C in 2024 and will reach 2°C around 2035.

Climate projections show:

i) the expected increase in average temperature during the winter in northern Croatia will be higher than in other parts of Croatia; the increase in average temperature in winter will be greater than in spring;
ii) summer temperatures will rise the most in the northern Adriatic, and in other parts of Croatia this increase will be somewhat milder;
iii) the number of frosty days (when the temperature is below −10°C) and snow cover in mountainous regions, such as Gorski Kotar, will decrease;
iv) the number of consecutive hot days in summer will increase, as will the number of hot nights.

Anomalies and changes in climate parameters (temperature, precipitation, wind speed) are associated with chronic and acute hazards (Table 1). The extent to which these hazards translate into sensitivity and exposure defines vulnerability for specific sectors, regions or stakeholders in Croatia (c.f. Beslik and Causevic, 2019). Coastal regions and islands, for instance, are facing increasing exposure to storms, waves, coastal flooding, and erosion. Acute hazards, such as cold or heat waves, floods, droughts, wildfires and storms, are becoming more common, and are geographically unevenly distributed (WMO, 2022). Areas with high temperatures are more susceptible to heatwaves, which pose risks to human health, infrastructure and ecosystems.

### 3.2 Impact of climate change on socio-economic transformation

The Intergovernmental Panel on Climate Change's Sixth Assessment Report (AR6) highlights the far-reaching implications of climate change for socio-economic development. The extent of these impacts is determined by both the magnitude of climate-related changes and the adaptive capacity of countries, regions, or stakeholders. The severity of projected outcomes differs across scenarios, geographic areas, and economic sectors. Effective implementation of global climate policies can reduce both exposure to climate hazards and vulnerability to their effects.

A growing body of research quantifies the economic consequences of climate change, highlighting both its direct impact on GDP and its role in deepening social and ecological vulnerabilities.

Nordhaus (1992), Dell et al. (2012), and Burke et al. (2015) find that a 1°C temperature increase reduces annual GDP by 1–2% in the medium run. Stern (2006) estimated that the cost of mitigation is about 1% of GDP—et relatively small to the costs and risks of climate change that will be avoided. Recent estimates are substantially higher. Bilal and Känzig. (2024) suggest that a 1°C increase in global temperature could lead to a 12% reduction in world GDP. There is increasing evidence that climate change exacerbates existing social inequalities. Initial inequalities cause disadvantaged groups to suffer disproportionately from the adverse effects of climate change, resulting in greater subsequent inequality (Islam and Winker, 2017).

In addition to damages and losses, climate change might threaten (i) survival and (ii) basic human needs (Huggel et al., 2022). The Dasgupta Review (Dasgupta, 2021) underscores the fundamental connection between biodiversity and economic prosperity, emphasizing that the degradation of natural ecosystems weakens economic resilience to climate shocks. It calls for the inclusion of natural capital in decision-making to promote sustainable development and mitigate the socio-economic risks associated with climate change.

The data on fatalities due to climate extreme shows that the climate already threatens survival. In the period 1980–2023, weather and climate extremes caused 910 fatalities in Croatia (EEA, 2024).

Beyond direct physical damage, climate change also affects human health and labor productivity. Deteriorating air quality contributes to higher disease prevalence, while increased heat stress, extreme weather events, and disruptions to economic activities reduce productivity. Rising temperatures lower workers' efficiency, particularly in sectors that rely on outdoor labor, such as agriculture and construction. According to the International Labor Organization (ILO), heat stress alone could result in a global loss of 2.2% of total working hours by 2030—equivalent to 80 million full-time jobs (Kjellstrom et al., 2019).

Furthermore, climate change influences the demand for specific skills and reshapes the composition of the workforce. The transition to a low-carbon economy necessitates the development of green technologies and sustainable practices, giving rise to new employment opportunities. These include, for example, roles in renewable energy, energy efficiency, environmental management, sustainable agriculture, circular economy sectors, and climate adaptation services. The World Economic Forum (WEF) highlights a growing disparity between the demand for green skills and their availability in the labor market. While global demand for such skills has increased by 40% since 2015, only 13% of the workforce currently possesses the necessary competencies, revealing a significant skills gap (World Economic Forum, 2023). This mismatch underscores the urgent need for comprehensive workforce development programmes and educational initiatives to equip workers with the qualifications required across a wide range of emerging green sectors. Simultaneously, certain industries are expected to face job losses due to climate policies and environmental change, making reskilling and transition support essential to ensure a just and equitable shift toward sustainable economic models.

In the context of the European Green Deal (EGD), impact assessments reveal that transitioning to a low-carbon economy presents both challenges and opportunities. While certain sectors may face economic restructuring, the overall strategy aims to promote sustainable growth, job creation, and improved public health. EGD seeks to mitigate the socio-economic risks associated with climate change, ensuring a just transition. EU-wide impact assessments primarily examine aggregate effects up to 2030, identifying the potential range of affected sectors and their dynamic transformations over time (Boehm and Kläffling, 2024).

A review of studies on the employment effects of the green transition (Table 6) shows broad agreement that, despite methodological differences, a low-carbon economy is likely to yield net job gains by 2030. The European Commission's broad modeling range reflects both uncertainty and policy sensitivity (European Commission, 2020), whereas CEDEFOP (2021) projects more robust growth based on favorable labor market dynamics and skill adaptation. Eurofound (2023) offers a cautiously optimistic scenario, emphasizing significant regional, occupational, and sectoral differences, and the importance of sector-specific factors such as construction and investment financing strategies. It also identifies the challenge of a crowding-out scenario: households and firms must cut other expenditures to finance energy and climate investments, which can have a negative overall impact on jobs. Overall, job quality improves, as measured by wages. However, most additional job growth, compared to the baseline, occurs in low- and middle-paid positions, primarily in the construction sector. Asikainen et al. (2021) provide a structural insight, indicating that, while fossil fuel sectors face steep declines, the overall employment impact may be contained due to their limited share of total jobs. Collectively, these findings reinforce the need for coordinated labor market policies and targeted reskilling efforts to ensure the green transition yields inclusive and equitable employment outcomes.

**Table 6:** Comparative employment impact of the green transition.

**Source**	**Employment impact by 2030**	**GDP impact by 2030**	**Distributional notes**
European Commission (2020)	−0.43% to +0.6%	−0.7% to +0.55%	Depends on model assumptions; broad aggregate estimate
CEDEFOP (2021)	+1.2% (≈2.5 million jobs)	Not specified	Baseline: pre-COVID skills forecast; strong upskilling emphasis
Eurofound (2023)	+0.1% (≈204,000 jobs)	Not specified	Significant sectoral/regional variation; construction key offset
Asikainen et al. (2021)	Sectoral declines (coal/oil/gas: −20.7% to −22%)	Not specified	High emission sectors = 8% of jobs, but 80% of emissions

Source: author.

More granular sectoral and national perspectives are provided in National Energy and Climate Plans (NECPs). The EU member states are required to prepare NECPs by the Governance Regulation (European Parliament and the Council, 2018). These plans link national policy frameworks to broader EU climate and energy targets. The EU member states typically use various economic models and impact assessment tools to analyse the connections between policy decisions and their effects on society and the economy. However, a meta-analysis of the current state of knowledge regarding the methodology and impact of selected policies and measures shows differences between ex-ante assessments and ex-post evaluations (Wachsmuth et al., 2023). This highlights the importance of robust, evidence-based methodologies to ensure the credibility and comparability of national climate policy evaluations.

Croatian NECP does not include assessment of the macroeconomic impact of the planned policies and measures nor has an adequate analytical basis to assess social impacts (European Commission, 2023a). This lack of analytical depth is particularly concerning given Croatia's high vulnerability to climate risks. The Notre Dame University's Global Adaptation Initiative (ND-GAIN) index summarizes a country's vulnerability to climate change and other global challenges in combination with its readiness to improve resilience. ND-GAIN framework breaks measure of vulnerability into exposure, sensitivity and adaptive capacity. Six life-supporting sectors are considered: food, water, health, ecosystem services, human habitat and infrastructure (Chen et al., 2024a).

In this context, based on ND-GAIN index, Croatia is amongst the most vulnerable upper-income countries (ranked 120th among 187 countries based on vulnerability component of the index), and the 63rd most ready country (Gain.nd.edu., 2024). With country index score 55.4, Croatia is ranked 55th. Among the EU member states, only Romania is ranked lower (University of Notre Dame, 2025). This high level of vulnerability is reflected in the substantial economic losses Croatia has incurred from weather and climate extremes over recent decades. Damage from weather and climate extremes in the period 1980–2023 is estimated at around €4,154 million, or €943 per person (EEA, 2024), which exceeds 0.25% of GDP per year. Only 2% of the damage was covered by insurance. This low insurance coverage further amplifies the socio-economic vulnerability to climate-related events. In contrast, Denmark and Norway insured more than half of claims (62% and 70% respectively, EEA, 2024). The comparison illustrates significant disparities in adaptive capacity and financial preparedness among European countries.

### 3.3 Population and labor market data and scenarios

According to the 2021 census, Croatia has 3.87 million inhabitants, representing a loss of almost 10% of its population compared to 2011. The average age in Croatia has increased by 10 years over the past 50 years, from 34 years in 1971 to 44.4 years in 2021. Only 14% of the population is in the age group 0–14 years (552 thousand), 63% of the total number (2.45 million) are of working age (15–64), and the share of the population aged 65 and over is 22% (851 thousand, DZS, 2022a). The active population in 2021 was 1.82 million, including 1.68 million employed and 130,000 (7.5%) unemployed (DZS, 2022b).

Despite significant demographic changes, national population projections for Croatia have not been updated since 2011. The projections prepared by Grizelj and Akrap (2011) include three variants—low, middle, and high. The low variant, representing the most conservative scenario at the time, projected a population of 4.3 million in 2021, 4.1 million in 2031, 3.9 million in 2041, and 3.7 million in 2051. However, the 2021 census recorded a population below these projections, indicating that depopulation is progressing at a faster rate than previously estimated.

International projections consistently indicate a continued population decline, though the projected rates and levels differ based on methodological approaches and assumptions (see Table 7). According to the UN (2024), Croatia's population was projected to reach 3.92 million in 2021, 3.87 million in 2024, 3.72 million in 2030, and 3.23 million by 2050. These figures suggest a slower decline compared to observed census trends.

**Table 7:** International and national population projections.

**Source**	**2021/2022**	**2024/2025**	**2030/2031**	**2040/2041**	**2050/2051**
UN (2024)	3.92 M	3.87 M (2024)	3.72 M	—	3.23 M
Eurostat (2023a)	3.86 M (2022)	—	3.70 M	—	3.30 M (depending on scenario 3.2–3.5)
World Bank (2024a)	3.87 M (2021)	3.81 M (2025)	3.69 M	—	3.20 M
Grizelj and Akrap (2011)—low variant	4.30 M	—	4.10 M (2031)	3.90 M (2041)	3.70 M (2051)

Sources: UN (2024), Eurostat (2023a), World Bank (2024b), and Grizelj and Akrap (2011).

Eurostat (2023a) estimated the population at 3.86 million in 2022, with further declines projected to 3.7 million by 2030 and 3.3 million by 2050. In addition to baseline projections, Eurostat conducted sensitivity analyses based on alternative demographic parameters (lower fertility, higher mortality, increased migration, reduced migration and no migration). These included projections for 2050 ranging from 3.2 million under scenarios of lower fertility, reduced migration, or no migration, to 3.5 million under higher mortality assumptions, and 3.4 million with increased migration. Eurostat also projected an increase in the median age, expected to reach 49.9 years by 2050 under the baseline scenario, indicating significant population aging.

The World Bank (2024b) reported Croatia's population at 3.87 million in 2021, with forecasts of 3.81 million in 2025, 3.69 million in 2030, and 3.2 million by 2050. These estimates are consistent with the results of the 2021 census. The World Bank dataset includes population projections by 5-year age groups, as well as by urban and rural residency and age dependency ratios. This level of demographic disaggregation enables further analysis of labor market trends and regional demographic dynamics, and was therefore used for scenario development in this study.

The World Bank's Population Estimates and Projections Database (World Bank, 2024b) reported Croatia's population at 3.87 million in 2021, with projections indicating a decline to 3.81 million in 2025, 3.69 million in 2030, and 3.2 million by 2050. These figures are consistent with the results of the 2021 census and align closely with Eurostat's projections. The World Bank projections include detailed disaggregation by age groups, urban and rural population, and age dependency ratios, making them suitable for use in labor market scenario development.

According to these projections, Croatia's population is expected to decline by 106,000 persons (~3%) between 2021 and 2026. The decline is more pronounced in the working-age population, which is projected to shrink by 107,000 individuals (4.4%) during the same period (Table 8). The dependency ratio is also expected to increase, rising from 56.6% in 2021 to 59.3% in 2026, primarily driven by a growing proportion of the population aged 65 and over. The old-age dependency ratio is projected to rise from 33.9% to 37.4% by 2026.

**Table 8:** Population estimate (2021) and population projections (2026, 2030, 2035, and 2050).

**Indicator**	**2021**	**2026**	**2030**	**2035**	**2050**
Population, in total, mil.	3.90	3.79	3.70	3.58	3.20
Decrease compared to 2021,%^*^		2.82	5.13	8.21	17.95
Population 15–64 years, mil.	2.49	2.38	2.29	2.18	1.81
Population decrease compared to 2021, %^*^		4.42	8.03	12.4	27.31
Share of population 15–64 years in total, %	63.9	62.8	61.9	60.9	56.6
Age dependency ratio, total, % of working-age population	56.6	59.27	61.7	64.3	76.8
Age-dependency ratio, old Dependents, over 65 years of age, % of working-age population	33.9	37.4	40.5	43.6	54.5
Urban population, % of total population	57.878	59.728	61.454	63.874	71.31
Rural population, % of total population	42.122	40.272	38.546	36.126	28.69
Urban population	2,256,663	2,266,000	2,275,000	2,287,000	2,280,000
Rural population	1,642,337	1,528,000	1,427,000	1,294,000	917,000

Source: World Bank (2024a) and authors' calculations. ^*^Indicates author's own calculations.

By 2030, the total population is projected to decline to 3.7 million, with the working-age population decreasing to 2.29 million. By 2050, the total population is expected to reach 3.2 million, and the working-age population is projected to fall to 1.89 million. This represents an overall population decline of ~11%, while the working-age population is expected to decline by 17% over the same period (Tables 8, 9).

**Table 9:** Labor market development scenarios.

**Scenario**	**Indicator**	**2021**	**2026**	**2030**	**2035**	**2050**
Scenario 1: a constant active population	The number of working-age population (16–64), mil.	2.49	2.38	2.29	2.18	1.81
	Active population, mil	1.816	1.816	1.816	1.816	1.816
	Activity rate	72.9%	76.2%	79.3%	83.3%	100%
	Employed (unemployment rate 7.5%), mil	1.68	1.68	1.68	1.68	1.68
Scenario 2: activity rate (73%), unemployment rate 5–10%	The number of working-age population (16–64), mil.	2.49	2.38	2.29	2.18	1.81
	Active population (activity rate 73%)	1.818	1.737	1.672	1.591	1.321
	Employed (unemployment rate 5%), mil	1.727	1.651	1.588	1.512	1.255
	Employed (unemployment rate 10%), mil	1.636	1.564	1.505	1.432	1.189

Source: author.

Depopulation is more pronounced in rural areas. The share of the urban population is projected to increase from 57.9% (2021) to 61.5% (2030) and 71.3% (2050; Table 8). These demographic changes raise concerns about future labor supply. To assess the potential size of the workforce, two scenarios were developed:

(i) Scenario 1 assumes constant active population and unemployment rate at 2021 level, that is, 1.816 million active population and 7.5% unemployment rate (i.e., 1.68 million employed persons in Croatia, DZS, 2021a);
(ii) Scenario 2 assumes a constant activity rate, at 2021 level (73%) and unemployment rate between 5 and 10%.

Scenario 1 implies a constant increase in the activity rate, reaching 100% in 2050 (Table 9). By 2050 Scenario 2 leads to a shrinking employment to 1.255–1.189 million (depending on the unemployment rate), a decline of up to 30% compared to 2021. In the same period, total population is projected to decrease by 18%. Limiting decline in employment to match the reduction in total population (i.e., 18% in 2050 compared to 2021) requires an increasing participation rate by 5.5 percentage points (under assumption of 5% unemployment rate), requiring the activation of an additional 190,000 persons. This necessitates greater participation and employment of women, as the male labor force participation rate is currently 10 percentage points higher than that of women, while the female unemployment rate exceeds the male unemployment rate by 1 percentage point (DZS, 2025).

Similarly, to limit employment reduction to 8% by 2035—matching population decline**—**it is necessary to almost double employment of workers over 65 years of age (from the current 31,000 to 60,000 workers) while keeping current activity rates in other age groups.

Migration is not explicitly modeled in the population projections; however, it represents an increasingly relevant demographic factor. In 2024, ~200,000 work permits were issued to foreign nationals, primarily for employment in the construction, transport, and tourism sectors, indicating a growing reliance on immigration to offset labor shortages.

### 3.4 GDP scenarios

According to Eurostat data for 2021, GDP per capita in Croatia at market prices (EUR 15,020) was at the level of 46% of the EU average (which amounts to EUR 32,430; Eurostat, 2022). Regional disparities are large and increasing. GDP per capita is at the level of 36% of the EU average in the counties of Eastern Croatia, and in Zagreb 118%. Also, there are increasing differences between urban and rural areas.

The scenarios for GDP (total and per capita) until 2050 are shown in Table 10. Maintaining GDP at current levels (EUR 58,285 million; DZS, 2022b), with depopulation shown in Table 8, means an increase in GDP per capita by 20%.

**Table 10:** GDP and GDP per capita scenarios.

**Scenario**	**Indicator**	**2021**	**2026**	**2030**	**2035**	**2050**
Stagnation of total GDP	Population, mil.	3.9	3.79	3.7	3.58	3.2
	GDP, total, million €	58,287	58,287	58,287	58,287	58,287
	GDP per capita, €	14,945	15,379	15,753	16,281	18,215
Reaching the EU average (GDP per capita)	Population, mil.	3.9	3.79	3.7	3.58	3.2
	GDP, % of the EU average	46	56	66	76	100
	GDP per capita, €	15,020	18,285	21,550	24,816	32,652

Source: author's projection.

The scenario of reaching the average GDP of the EU per capita by 2050, assumes reaching 66% of EU GDP in 2030 and 100% in 2050 (Table 10).^1^

As working force is projected to shrink, both scenarios require improvement in labor productivity.

### 3.5 Sector-specific vulnerabilities and opportunities

Identification of sector-specific vulnerabilities and opportunities depends on current economic structure and potential for its development. Criteria for selection of sectors are based on their economic significance (measured by contribution to the GDP and employment), potential for GHG emission reduction, vulnerability to climate change and growth opportunities driven by the implementation of climate policies. Economic significance is analyzed based on 4-year average (2020–2023), while year-to-year variations in employment and GDP contribution were also considered to assess sector-specific vulnerabilities and adaptive capacity.

The top five sectors contributing to Croatia's GDP (4-year average, in current prices) are manufacturing (12% of the GDP in 2019–2022 period), wholesale and retail trade (9.92%), real estate activities (7.68%), public administration and defense (5.77%) and construction (4.99%, Table 11).

**Table 11:** Gross value added according to production approach, 2019–2022, current prices , %.

**Sectors according to national classification of economic activities**		**2019**.	**2020**.	**2021**.	**2022**.	**Average 2019–2022**	**Average 2020–2022**
**Code**	**Description**						
A	Agriculture, forestry and fisheries	2,9	3,1	3,4	3,4	3,18	3,29
B	Mining & Quarrying	0,3	0,2	0,2	0,2	0,22	0,19
C	Manufacturing	12,0	12,2	11,7	12,3	12,02	12,03
D	Electricity, gas, steam and air conditioning supply	1.8	1.3	1.4	1.4	1.48	1.38
E	Water supply; wastewater removal, waste management and environmental remediation activities	1.0	1.4	1.3	1.3	1.25	1.33
F	Civil engineering	4.5	5.0	5.0	5.0	4.86	4.99
G	Wholesale and retail trade; repair of motor vehicles and motorcycles	10.0	10.1	9.8	9.8	9.92	9.89
H	Transportation and storage	4.2	4.4	4.3	4.7	4.43	4.50
I	Accommodation and food service activities	5.1	2.5	4.2	5.7	4.37	4.13
J	Information & Communications	3.9	4.9	4.8	5.2	4.71	4.97
K	Financial and insurance activities	4.5	4.3	4.4	4.0	4.28	4.22
L	Real Estate Business	7.5	8.2	7.6	7.5	7.68	7.75
M	Professional, scientific and technical activities	4.7	4.6	4.3	4.6	4.54	4.50
N	Administrative and support service activities	2.0	1.7	1.8	1.8	1.82	1.77
O	Public Administration and Defence; Compulsory social security	5.7	6.5	5.8	5.2	5.77	5.80
P	Education	4.2	4.6	4.4	4.1	4.34	4.38
Q	Health care and social welfare activities	4.2	4.8	4.7	4.4	4.52	4.63
R	Arts, entertainment and recreation	1.7	1.5	1.4	1.4	1.52	1.45
S	Other service activities	1.5	1.5	1.5	1.4	1.47	1.44
T	Activities of households as employers; activities of households producing different goods and providing different services for their own needs	0.0	0.0	0.0	0.0	0.03	0.03
U	Activities of extraterritorial organizations and bodies	-	-	-	-		

Source: author based on https://podaci.dzs.hr/hr/podaci/bdp-i-nacionalni-racuni/godisnji-bdp/.

In terms of employment, the top five sectors are: wholesale and retail trade, education, human health and social work activities, construction, public administration and defense (DZS, 2021b, 2024).

The climate change mitigation policies focus on sectors with highest emissions, i.e., the largest potential for decarbonization. In Croatia, these sectors are energy, agriculture, buildings, transport, industry and waste management (Ministry of Economy and Sustainable Development, 2021, 2023).

In parallel, adaptation strategies highlight different sectoral concerns. Croatia's Adaptation Strategy identifies the most vulnerable sectors: water resources, agriculture, forestry, fisheries and aquaculture, energy, tourism, biodiversity, spatial planning and disaster risk management (Croatian Parliament, 2020).

Literature review reveals that climate policies and climate change provide opportunities for sectoral and regional growth. These include both established sectors, in which new services are emerging (e.g., renewables or electromobility), and entirely new sectors linked to innovation.

The renewable energy sector has experienced substantial growth, driven by advancements in technologies such as solar and wind power. According to the International Renewable Energy Agency (IRENA), the renewable energy sector employed 16.2 million people globally in 2023, marking an 18% increase from 13.7 million in 2022 (IRENA and ILO, 2024). In addition to traditional renewable energy sources, emerging fields like agrivoltaics—combining agriculture with photovoltaic energy production—present new avenues for growth. Agrivoltaics systems can improve crop yields and water efficiency (Pandey et al., 2025).

Development of new market and governance models (e.g., distributed energy, storage and energy communities) provide potential for development of new products and services and relies on innovation. In Croatia this is a challenge, given the limited innovation, and availability of a highly educated workforce in Croatia. Innovation score (0.049) is the lowest score in readiness dimension of the ND-Gain index for Croatia. This score is measure of the number of patent applications, by residents per capita. Only 35% of the Croatian population has tertiary education, which is among the lowest shares in the EU and represents 76.0% of the EU average. Only 4% of the population engages in lifelong learning, which is among the lowest across EU (45.8% of the EU average; EFIS Centre Technopolis Group and OldContinent, 2024).

Applying the multi-criteria framework outlined in the methodology, following key sectors are analyzed in more details: (i) energy (notable for high emissions, vulnerability and high growth potential), (ii) agriculture (notable for high emissions, vulnerability and high growth potential), (iii) tourism (vulnerability and relevance for Croatian economy and employment), (iv) construction (relevance for mitigation, adaptation and employment) and (v) health (relevant for employment and adaptation, due to the aging of population; Table 12).

**Table 12:** Categorization of economic sectors.

**The biggest contribution to the GDP**	**The biggest contribution to employment**	**Sectors with the highest emissions (decarbonisation potential)**	**The most vulnerable sectors (loss and damage due to climate change)**	**Sectors with growth potential (possible positive effects)^a^**
• Manufacturing • Wholesale and retail trade • Real estate activities • Public administration and defense • Construction	• Manufacturing • Wholesale and retail trade • Education • Human health and social work activities • Construction, • Public administration and defense	• Transport • Energy (fossil fuels) • Manufacturing • Buildings • Agriculture • Waste management	• Water resources^b^ • Energy (e.g., hydropower) • Agriculture, forestry, fisheries • Tourism • Health	• Energy (renewables, solar energy, wind energy, biofuels) • Climate-neutral mobility (electric cars, public transport, green hydrogen) • Use of sustainable materials • Carbon capture and storage • Forestry, agriculture (sinkholes) • Emerging Sectors (Innovation)

Source: author.

^a^Here, possible positive effects mean the possibility of creating new jobs, but it does not necessarily mean that the overall social effects are positive. For example, the need for firefighters and emergency services will increase, which increases the pressure on public sector financing. The lack of job security in these sectors could have significantly greater negative consequences, but these have not been analysed or quantified in more detail here.

^b^Water resources are not usually categorized as a sector. The categorization taken here is from the Climate Change Adaptation Strategy.

#### 3.5.1 Energy

Energy sector is the biggest source of greenhouse gas emissions in Croatia (Ministry of Economy and Sustainable Development, 2021, 2023). It is dominated by large, incumbent companies that are partially or fully owned by the state (e.g., HEP Group, INA, Janaf, Plinacro). Croatia relies dominantly on (imported) carbon-based fuels. Import dependency is roughly 50% and the share of fossil fuels in gross available energy accounts for 70% (Eurostat, 2024; Boromisa, 2022; IRENA, 2024). Coal phase out is announced for 2033, while gas capacities (including LNG terminal) are increasing. Expansion of gas infrastructure is considered a national strategic project (Government of the Republic of Croatia, 2025).

There is a significant potential and interest for investment in new and renewable energy (photovoltaics and wind).^2^

Decarbonization and decentralization in the energy sector enable the development of new business models, the involvement of small entrepreneurs and citizens in the energy transition. This could limit exposure to rising energy prices, support achieving emission reduction targets.

The lack of clear priorities and persistent barriers to sector reform hinder decarbonization efforts, which could otherwise drive modernization, decentralization, and improved corporate governance within incumbent companies—ultimately reducing opportunities for corruption. However, high-profile corruption cases in the energy sector, including the sentencing of a former prime minister to 6 years in prison and ongoing investigations involving a former secretary of state and several high-ranking officials, have further exacerbated concerns. Coupled with Croatia's high Corruption Perception Index (Croatia ranked 63^rd^ of 200 countries in 2024, Transparency International, 2024), these factors deter non-speculative investments and contribute to administrative and market barriers, including also long equipment delivery times and a shortage of skilled installers. These challenges limit the scale of investment and slow the pace of sector transformation (Ministry of Economy and Sustainable Development, 2022a,b). According to the Ministry of Physical Planning, Construction, and State Property, while Croatia has 705 certified photovoltaic system installers, there are none for solar thermal systems, small biomass boilers and stoves, shallow geothermal systems, or heat pumps (Ministry of Physical Planning, Construction and State Property, 2025).

Climate change already affects energy consumption and capacity of the energy sector to meet demand. During the period from 2010 to 2025, peak electricity demand in Croatia has gradually shifted from winter to summer months. This trend is linked to increased use of air conditioning during heatwaves and the intense tourist season. The climate change will further increase cooling needs (a higher number of cooling-days). While there is a significant potential to produce solar energy in the summer, in summer 2024 Croatia imported about one-quarter of its required electricity.

Share of hydropower in electricity production (38% in 2022, IRENA, 2024) makes power sector very vulnerable to climate change. Projected decrease in participation is likely to have negative impact on generation. In addition, output of thermal plants will be limited by availability of water for cooling. For example, drought in 2003 caused damage between 63 and 96 million euros to the energy sector (Croatian Parliament, 2020). Losses and damages from extreme events limit the potential for investment in adaptation measures (e.g., systems to increase the reliability of forecasts).

EU-level analyses show that the clean energy sector contributes to the creation of new jobs (mainly for low- and medium-skilled workers) and that electrification of the economy could generate 2.0% of additional jobs in the electricity sector (Asikainen et al., 2021).

In Croatia, the Low-Carbon Strategy envisions investments of €4–6 billion in renewable energy sources by 2030, with the potential to create 50,000 new jobs (Official Gazette 63/2021). However, this presents a significant challenge, as projections indicate that by 2030, the working-age population will decline by 190,000 compared to 2020—nearly double the current number of unemployed persons. As of the latest data, Croatia has 96,578 registered unemployed individuals (HZZ, 2025), highlighting a potential labor shortage that could hinder the implementation of existing policies.

Energy is moderately to highly exposed, particularly under SSP3-7.0, due to increased cooling demand, reduced hydropower availability, and climate-induced demand shifts. It is highly sensitive, given its dependence on imports and vulnerability of hydropower systems. The sector's vulnerability is high, and resilience is moderately low, as structural reform and diversification efforts are progressing slowly and remain constrained by governance challenges (Table 13).

**Table 13:** Sector-specific exposure, sensitivity, vulnerability and adaptive capacity.

**Sector**	**Exposure (SSP2-4.5/SSP3-7.0)**	**Sensitivity**	**Vulnerability (overall)**	**Resilience**
**Agriculture**	High (↑ temperature, ↓ precipitation, ↑ extreme events)	Very High (yield loss, small farms, aging workforce)	Very high	Very low (high vulnerability + poor insurance, low R&D, low mechanization)
**Tourism**	High (↑ heatwaves, sea-level rise, extreme weather)	High (seasonal dependency, infrastructure strain)	High	Medium low (high vulnerability but national strategy exists, uneven implementation)
**Energy**	Medium–High (↑ cooling demand, ↓ hydropower capacity)	High (import reliance, hydro-dependency)	High	Moderate low (high vulnerability + slow diversification and reform)
**Construction**	Medium (heat, material availability, storm risk)	Medium (labor intensity, safety, economic linkages)	Medium–high	Moderate low (medium high vulnerability, labor shortages, high informality)
**Health**	Medium (heatwaves, air pollution, vector-borne diseases)	High (aging population, uneven rural access)	High	Low (infrastructure gaps, workforce shortage, increased demand)

Source: author.

#### 3.5.2 Construction

Construction employs around 105,000 people (6% employment) and accounts for 5% of GDP (Tables 7, 8). Undeclared work and insufficient safety at work are still present. Average salaries are about 20% lower than the Croatian average. In 2023 average monthly gross earnings were EUR 1,282, compared to national average EUR 1,584. The sector faces a shortage of workers. According to the data of the Ministry of the Interior for 2021, every fifth employee in construction in Croatia is a foreigner (according to Marić, 2021).

Based on estimates by the Buildings Performance Institute Europe (BPIE, 2020), an average investment of EUR 1 million in energy renovation generates 18 new jobs across the European Union. In Croatia, this job creation potential is significantly higher, estimated at 29 jobs per EUR 1 million invested, including 11.5 direct, 9.5 indirect, and 8 induced jobs. According to the Long-Term Strategy for the Renovation of the National Building Stock by 2050, Croatia plans to invest EUR 9.5 billion in building renovation by 2030 (Government of the Republic of Croatia, 2020). Based on the BPIE employment multiplier, this investment is projected to result in the creation of ~275,500 jobs, of which 109,250 are expected to be direct jobs. However, financial constraints and a shortage of skilled labor remain critical barriers to the effective implementation of these climate-related investment plans. The strategy also includes the reconstruction of buildings damaged in the 2020 earthquakes. However, as noted by the Prime Minister, reconstruction efforts remained “unrealistically slow” until 2023 (Plenković, 2023). Furthermore, the legal framework governing investments in green energy remains underdeveloped (Ministry of Economy and Sustainable Development, 2022a). The persistent shortage of qualified workers continues to impede the progress of both energy renovation initiatives and public infrastructure projects.

National Energy and Climate Plan (NECP) further highlights the workforce challenge, estimating that the implementation of planned measures will require an additional 23,061 construction workers by 2030, and a total of 47,747 by 2050. Without strategic efforts to address labor shortages—including workforce training and the international recruitment of skilled workers—the successful implementation of climate and energy policies remains uncertain.

Construction experiences medium exposure, primarily through temperature extremes, materials availability, and storm-related risks. Its sensitivity is moderate, as the sector is labor-intensive and faces persistent issues with worker safety and regulatory enforcement. The overall vulnerability is medium to high, and resilience is moderately low, due to labor shortages, a high degree of informality, and implementation challenges for climate-aligned building policies (Table 13).

#### 3.5.3 Agriculture, forestry and fisheries

In Croatia, agriculture accounts for 3% of GDP and 6.7% of employment, both below the EU averages of 4.7% and 8%, respectively. The sector has experienced annual declines of 3.7% in gross output and 4.3%, in value-added. It is characterized by small-scale, fragmented farms, with 70% of farms smaller than 5 hectares. Production remains predominantly low-value, with limited integration into global value chains. The insurance is insufficiently used (Croatian Parliament, 2022; Ministry of Agriculture, 2020). Labor vulnerabilities compound these issues: agricultural wages lag 20% below the national average (EUR 1,358 vs. EUR 1,584 in 2023), with over 50,000 workers reliant on seasonal or informal employment, often without contracts or protective equipment (Badanjak, 2021). Aging farmers, rural depopulation, and land abandonment -projected to reach 11% at the EU level by 2030 (Perpiña Castillo et al., 2018; JRC, 2018) further threaten modernization. Climate change exacerbates risks, with rising temperatures, droughts, and extreme weather shortening growing seasons, altering phenology, and increasing water demand. Projected yield losses of 10–60% (up to 90% regionally) without irrigation, and an overall 3–8% decline by 2050 under SSP2-4.5 (Croatian Parliament, 2020), highlight acute vulnerabilities, particularly for cereals and maize, despite potential winter crop benefits.

Adaptation measures include investments in irrigation, controlled-environment agriculture, and the adoption of climate-resilient crops. Improved education and knowledge transfer are critical to enhancing farmers' ability to adopt resilient species, new technologies, and advanced management practices (Munech et al., 2022). Indicators of fertilizer, pesticide, irrigation, and mechanization use further highlight Croatia's vulnerability to climate change.

Forests cover ~50% of Croatia's land area. Climate change is increasing the frequency and intensity of wildfires, windthrows, ice storms, floods, and pest outbreaks, leading to rising annual losses. These trends threaten the forest sector's ability to act as a carbon sink and provide essential ecosystem services, such as water and air purification, flood and erosion protection, and climate regulation (Croatian Forests, 2022). At the same time, climate-related risks are increasing demand for forest monitoring, protection, and management services, affecting employment structures in forestry.

Climate change is also reshaping the economic viability of fisheries. Rising Adriatic Sea temperatures are expected to drive marine species migration into deeper and northern waters, increase the presence of invasive alien species, and reduce or eliminate certain native fish populations. These changes necessitate shifts in fishing practices and species selection for aquaculture. While warming conditions may benefit tuna and fennel farming, they pose challenges for sea bass and oyster farming.

Additionally, reduced freshwater availability may constrain inland aquaculture, though higher temperatures could accelerate fish growth cycles. Ocean acidification is another major concern, particularly for shellfish farming, potentially threatening production in specific coastal areas.

Agriculture, fisheries and forestry are highly exposed under both scenarios—particularly SSP3-7.0—due to increased temperatures, reduced precipitation, and more frequent extreme events. Agriculture demonstrates very high sensitivity, stemming from structural issues such as small farm size, an aging workforce, and low-value production. Consequently, its overall vulnerability is very high, and resilience is very low, given inadequate insurance coverage, low levels of mechanization, and limited investment in R&D.

Fisheries face high exposure, driven by warming seas, ocean acidification, and invasive species, all of which are exacerbated under SSP3-7.0. With medium to high sensitivity, the sector is challenged by stock redistribution and aquaculture losses. Vulnerability is high, while resilience is low to moderate, as early-stage adaptive responses are emerging but are not yet robust or widely implemented. Forestry has moderate sensitivity, as ecosystem degradation and fire risk directly affect forest function. The sector's vulnerability is high, with low resilience, hampered by an aging workforce and insufficient investment in monitoring and adaptive forest management (Table 13).

#### 3.5.4 Tourism

Tourism is Croatia's leading economic sector, contributing 19.5% to national GDP in 2022—the highest share among European countries (Ministry of Tourism and Sports, 2023b). However, the sector is highly sensitive to climate change, as shifts in local climatic conditions may alter the length of the tourist season and influence the spatial and temporal distribution of tourism activities (EEA, 2017). In Croatia, these changes are expected to lead to both geographic and seasonal shifts in tourism patterns.

Rising temperatures, increased UV radiation, and extreme weather events are projected to reduce tourist demand along the coast during the peak summer months. Additionally, the Adriatic Sea is expected to rise by 16–23 cm by the mid-21st century, depending on the emissions scenario (World Bank, 2022). Due to the low-lying nature of the coastline, this poses significant risks to coastal and island municipalities, including key tourist destinations such as Cres, Mali and Veli Lošinj, Krk, Rab, Krapanj, Vela Luka, Nin, Trogir, Ston, Pula, Split, and Zadar. The increased frequency of coastal flooding could lead to biodiversity loss and damage to cultural heritage sites, thereby reducing the attractiveness of established tourist locations. Furthermore, climate-induced pressures on infrastructure—including water supply, wastewater management, solid waste disposal, beach facilities, and accommodation complexes—may further diminish the sector's resilience and appeal.

The tourism industry is already experiencing labor shortages, both in terms of workforce availability and skill levels. In 2019, there was a deficit of 42,927 workers in the sector, with existing employees often lacking the necessary qualifications to meet labor market demands (Ministry of Tourism and Sports, 2023a). Between 2009 and 2019, the share of employment in accommodation, food services, and hospitality increased from 5.8% to 8.2%, with the seasonal nature of tourism driving precarious employment. Seasonal workers typically face lower wages, longer working hours, fewer training opportunities, limited career progression, and job insecurity compared to permanent workers (c.f. Fearden, 1985). Climate change-related economic losses—resulting from declining destination attractiveness and infrastructure damage—could exacerbate these challenges by reducing opportunities for permanent employment and intensifying labor market instability.

Recognizing the interconnections between tourism and climate change, Croatia's Sustainable Tourism Strategy until 2030 (Ministry of Tourism and Sports, 2023a) emphasizes the need for a transition toward more sustainable tourism practices. A significant aspect of this transition is the shift toward more sustainable modes of tourist transportation, both to and within the country. Currently, 60% of tourists travel to Croatia by car and 20% by air—both associated with high greenhouse gas emissions. Advancing sustainability in tourism thus necessitates substantial investment in low-carbon transport infrastructure, including the expansion of rail networks, the installation of charging stations for electric and hydrogen vehicles, improved public transport connectivity, and the development of cycling infrastructure. These interventions would not only lower greenhouse gas emissions but also strengthen Croatia's positioning as a sustainable and climate-resilient tourism destination.

Tourism faces high exposure to climate risks across both scenarios, including rising temperatures, sea-level rise, and increased frequency of extreme weather events. It shows high sensitivity, due to its seasonal nature and infrastructure dependence. Its vulnerability is high, although resilience is moderately low: while a national tourism strategy exists to address climate adaptation, implementation remains inconsistent and uneven across regions (Table 13).

#### 3.5.5 Health

Climate change exacerbates existing public health challenges, introduces new risks, and exposes weaknesses within the healthcare system (c.f. Harlan et al., 2013). An aging population and a relatively low number of medical professionals (measured in doctors and nurses per 1,000 inhabitants) further heighten climate-related health risks. These challenges are particularly pronounced in rural and island communities, where access to healthcare services is already limited. Strengthening health infrastructure to withstand climate impacts—through adequate heating, cooling, and ventilation—can enhance the efficiency of healthcare workers and facilitate faster patient recovery (cf. BPIE, 2018).

Data on extreme weather events and related health outcomes indicate a rising mortality rate during heat waves. For example, in August 2003, mortality in Croatia was 4% higher due to heatstroke (Croatian Parliament, 2020). Similarly, during the late June 2021 heatwave, daily maximum temperatures in Zagreb reached 35–38°C, leading to a 20% increase in emergency medical interventions related to heart attacks and collapses [DHMZ, as reported to WMO (2022)].

Temperature extremes—both heatwaves and cold spells—worsen chronic conditions and contribute to excess mortality. Prolonged exposure to high temperatures increases the incidence of acute and chronic illnesses, including cardiovascular and respiratory diseases, and raises mortality rates. Warmer temperatures also facilitate the spread of vector-borne diseases and intensify respiratory conditions due to higher pollen concentrations. Furthermore, unmitigated climate change poses risks to mental health, potentially leading to increased cases of anxiety, depression, and even suicidality, highlighting the growing demand for mental health services (MuŽinić Marinić, 2023).

Other environmental hazards linked to climate change also contribute to adverse health outcomes. Air pollution from forest fires exacerbates respiratory and cardiovascular diseases, while floods increase the risk of injury and the spread of infectious diseases. Both floods and droughts are associated with higher rates of anxiety, depression, and post-traumatic stress disorder. Damage to healthcare infrastructure can further limit or completely disrupt access to medical services. These health impacts can, in turn, lead to behavioral changes, reduced productivity, and temporary or permanent work incapacity, while placing additional strain on healthcare professionals through increased workloads and challenging working conditions.

Despite the increasing risks posed by climate change, national policy documents—namely the National Health Development Plan (2021–2027) and its accompanying Action Plan—remain primarily focused on issues such as working conditions, remuneration, and career development. These documents largely overlook essential climate adaptation and mitigation measures, including the need for adequate heating, cooling, and ventilation systems in healthcare facilities, or the transition to electric and alternative-fuel vehicles within healthcare services. Integrating climate resilience into health policy is imperative to protecting public health in the context of a changing climate.

Health is moderately exposed, particularly under SSP3-7.0, to climate stressors such as heatwaves, air pollution, and the spread of vector-borne diseases. It shows high sensitivity, driven by Croatia's aging population and unequal access to healthcare, especially in rural areas. As a result, vulnerability is high, and resilience is low, constrained by insufficient infrastructure adaptation, workforce limitations, and rising demand for services under climate pressure (Table 13).

## 4 Discussion

Climate change affects all sectors and regions. Global estimates suggest that a 1°C increase in average temperature may reduce global GDP by 1–2%, with worst-case projections indicating potential losses of up to 12%. In addition, the probability of extreme weather events is projected to increase by a factor of 4.1 per decade if global warming reaches 1.5°C.

However, the intensity of its impacts and their socio-economic consequences vary considerably depending on levels of exposure, sensitivity, and vulnerability. The unmitigated effects are expected to disproportionately affect the most vulnerable populations and regions, particularly those with limited adaptive capacity.

Conversely, the transition to a low-carbon economy presents opportunities for net employment gains and the potential for economic transformation aligned with environmental sustainability. While some jobs will disappear, others will emerge—particularly in technology and green industries.

Croatia ranks among the most climate-exposed countries within the European Union, while simultaneously exhibiting some of the lowest levels of climate resilience. Evidence from the Notre Dame Global Adaptation Initiative (ND-GAIN) index and the European Environment Agency (EEA) suggests that this vulnerability results from the intersection of pronounced environmental exposure and significant socio-economic constraints, including limitations in adaptive capacity and institutional preparedness. Empirical climate data demonstrate that anomalies in average surface air temperatures have already reached an increase of 1°C relative to pre-industrial levels. Climate projections further suggest that the 1.5°C threshold may be crossed as early as 2024, with global mean temperature anomalies potentially reaching 2°C by ~2035.

Croatia has exceptionally low levels of climate-related insurance coverage and suffers from structural weaknesses in agricultural innovation and productivity. These limitations reduce its capacity to recover from climate-induced disruptions and constrain both long-term resilience and the ability to leverage climate adaptation as a development opportunity. Current climate policies tend to address these challenges primarily through infrastructure investment. For example, in Croatia, planned investments in building renovation are projected to create ~275,500 jobs by 2030, including 109,250 direct positions, based on employment multipliers. This infrastructure-driven approach is also reflected in broader national development objectives. National policy documents set the goal of converging with the EU average GDP per capita. However, scenario analysis suggests that achieving this by 2050 would require a doubling of current GDP per capita levels. During the same period, Croatia's total population is projected to decline by 18% and the working-age population by 27%. The age dependency ratio is expected to increase by 10 percentage points, and the old-age dependency ratio by 20 percentage points. These trends imply that substantial productivity gains will be needed to close the development gap and to compensate for long-term labor force contraction.

Preserving current GDP levels alone would require a 20% increase in GDP per capita, due to the accelerated decline of the working-age population relative to the total population. This puts further pressure on labor productivity and long-term growth.

Demographic decline poses a structural constraint on labor supply and may undermine Croatia's capacity to implement climate measures. This is especially evident in the construction sector, which already faces labor shortages and depends heavily on foreign workers. Although migration is not explicitly included in national workforce projections, ~200,000 work permits were issued to foreign nationals in 2024, indicating an increasing reliance on migrant labor to address immediate needs.

These workforce challenges could intensify the impacts of climate change by limiting implementation capacity and delaying adaptation efforts. As with other vulnerabilities, the effects are likely to disproportionately harm populations with limited adaptive capacity. Croatia's labor force is also less educated than the EU average. Only 35% of the population holds a tertiary degree—equivalent to just 76% of the EU average. Moreover, only 4% of adults participate in lifelong learning, limiting workforce adaptability (EFIS Centre Technopolis Group and OldContinent, 2024). Innovation output is similarly low, with only 15.7 patent applications per million inhabitants, compared to the EU average of 65.7 (European Commission, 2023b). Croatia invests just 1.27% of its GDP in research and development, significantly below the EU average of 2.3% (Eurostat, 2023b). These figures highlight that limited human capital and weak innovation capacity constrain both economic convergence and climate resilience.

The convergence objective is further complicated by the omission of macroeconomic and social impact assessments in Croatia's climate-related policy documents and action plans. These assessments are lacking both as foundational background studies and as integrated elements of policy design. Their omission reflects a broader disconnect between climate policy formulation and the socio-economic context—marked by depopulation, population aging, and heightened exposure to climate risks.

To address these constraints, redirecting investment from infrastructure toward skills development, research, and innovation is necessary. As the labor market evolves—with some jobs disappearing and others emerging—new opportunities will arise, particularly for technologically skilled workers and innovators. Yet investment in education and training, research and development, and technological advancement remains limited. Greater emphasis on these areas, together with increased women's labor force participation and integration of foreign workers would help alleviate labor shortages, reduce social exclusion and poverty, and strengthen macroeconomic performance (cf. Office for Gender Equality of the Government of the Republic of Croatia, 2022).

Increased exposure to climate extremes is expected to result in escalating economic damages. Between 1980 and 2023, the average annual losses attributed to climate-related events amounted to roughly 0.25% of national GDP. As temperature anomalies approach 1.5°C, the frequency and intensity of such events are projected to increase significantly—by a factor of 4.1—suggesting that annual damages could exceed 2% of GDP by 2030. This estimate does not account for the indirect costs of acute hazards—such as productivity losses or public health impacts—nor include damages resulting from chronic hazards.

The Croatian case highlights the importance of integrating horizontal enabling measures into climate policy design. A persistent methodological gap is the lack of incorporation of socio-demographic dynamics—including aging, depopulation, and regional disparities—into national climate strategies. Without this integration, there is a risk that policy ambitions will be misaligned with actual implementation capacity, particularly in countries facing structural labor market constraints. Addressing this gap requires coordinated investment in education, reskilling, and social protection systems, as well as territorially sensitive approaches that account for both climate exposure and socio-economic vulnerability.

The relevance of different sectors to climate-resilient development is shaped by a combination of economic structure and climate risk. Sectors are identified based on their contribution to GDP and employment, vulnerability to climate impacts, and potential to support a low-carbon transition. Based on these criteria, five sectors—tourism, energy, construction, agriculture, and health—are central to Croatia's climate-resilient development.

Tourism accounts for ~20% of Croatia's GDP. The sector is highly sensitive to changing weather patterns and seasonal variability. It also faces a chronic workforce shortage. Climate risks further threaten its long-term viability and place additional strain on an already limited labor supply.

Agriculture in Croatia is increasingly exposed to climate-related stressors. These include drought, heatwaves, and soil degradation. Crop yields are projected to decline by 3–8% by 2050. The sector plays a key role in regions that already lag behind the national average in economic performance and employment. These areas are also affected by precarious work and depopulation. Building a more resilient agricultural system will require substantial innovation and investment. However, the sector faces a shortage of skilled labor and limited opportunities for workforce development. These constraints are intensified by rural depopulation and a weak innovation ecosystem. Together, they limit the sector's adaptive capacity and long-term sustainability.

The energy sector also faces growing challenges. Rising demand increases the risk of climate-induced supply disruptions. Decarbonisation will require accelerated investment in renewable energy infrastructure. However, this transition is hindered by governance issues and a shortage of construction workers.

Construction is essential for both climate mitigation and adaptation. It plays a central role in energy-efficient retrofitting and the development of resilient infrastructure. Yet the sector suffers from persistent labor shortages and poor working conditions. It also shows limited adoption of adaptive building technologies. Given Croatia's increasing exposure to heatwaves, flooding, and coastal erosion, technological innovation in construction is essential for reducing emissions and enhancing resilience.

Healthcare is gaining importance as climate change increases health risks—such as those from heatwaves, air pollution, and climate-sensitive diseases—while an aging population further intensifies pressure on the health system.

Sectoral analyses show that climate-resilient development is a socio-economic challenge. Climate-related pressures (rising temperatures, extreme weather, and biodiversity loss) already disrupt economic activity in these areas. At the same time, a shrinking and aging workforce reduces the capacity to respond effectively. Regional disparities further limit implementation, especially in already vulnerable areas. These dynamics form a reinforcing feedback loop: climate change weakens economic stability, while demographic decline constrains adaptation and recovery.

Projections under medium (SSP2-4.5) and baseline (SSP3-7.0) scenarios confirm that labor shortages, skill mismatches, and regulatory barriers undermine Croatia's adaptive capacity. In climate-sensitive sectors, extreme events can lead to short-term job losses. Medium-term constraints include limited access to finance, poor working conditions, and declining entrepreneurial activity. Long-term risks involve an accelerated decline in workforce availability, growth in informal and precarious employment, and reduced innovation and investment (Tables 13, 14).

**Table 14:** Sector-specific dynamics of changes, medium (SSP2-4.5) and basic (SSP3-7.0).

**Time horizon**	**Most vulnerable sectors**		**Sectors with growth potential**		**High emissions sectors**	
	**Medium scenario (SSP2-4.5)**	**Baseline scenario (SSP3-7.0)**	**Medium scenario (SSP2-4.5)**	**Baseline scenario (SSP3-7.0)**	**SSP2-4.5 (Medium Scenario)**	**High emissions sectors SSP3-7.0 (Baseline Scenario)**
Short-term (until 2027)	Exposure to climate extremes causes damage • Programmes for the development of new skills, reskilling and upskilling are launched	Exposure to climate extremes causes damage • Job losses due to financial losses and the damage of climate change	Limited use of opportunities to develop new skills • A slight increase in existing opportunities	Regulatory barriers are limiting the development of new products and services. • Opening up opportunities for the development of new skills, upskilling and retraining	No significant changes	Limited use of opportunities to develop new skills
Medium term (until 2030)	Restructuring and use of collateral • Investments in resilient infrastructure	Investing in workers' skills • Investments in working conditions • Start of development of adaptation measures around 2030	Lack of financial capacity (due to damages) and workers limits the implementation of adaptation measures • An increase in informal work. • Losses and damages lead to a reduction in the number of entrepreneurs/ employers, and consequently the number of workers • deterioration of working conditions	Increase in opportunities (agroforestry, sustainable use of forests and timber products) • Increasing innovation and related jobs	A slight increase in opportunities, a decrease in the number of employees in line with demographic trends • Administrative restriction for certain activities. • Emphasized requirements for emission reductions	The beginning of restructuring • The dynamics of the closure of existing jobs follows demographic trends • There are no significant negative effects on workers. • Beginning of change around 2030. • Slow and partial restructuring • The dynamics of job cuts are faster than demographic trends
Long term (by 2050)	Implementation of adaptation measures, restructuring of the sector • Fewer workers, different skills.	Intensification of negative effects observed in the medium term • Unfavorable working conditions (more pronounced climate change), major damages and losses • Reducing the number of workers faster than demographic trends	Inability to recruit new workers, skills mismatch • Dependence on public funds/state aid • Intensification of medium-term effects	Shortage of workers • Development of emergency services (temporary housing, relocation)	Insufficient number of workers creates pressures related to working conditions (overtime, restrictions on the use of annual leave, etc.)	Labor shortages and skills mismatches are hampering the transformation of the labor market. • Gradual shutdown in accordance with the expected lifetime of the plant

Source: author.

Immediate action is needed to address these constraints and enable climate-resilient development. Policies must respond to demographic decline, labor shortages, and overdependence on tourism. At the same time, they must support the transformation of high-emission sectors such as energy, industry, transport, and buildings. Life-supporting sectors—including water, health, ecosystems, and infrastructure—also require targeted investment and institutional support.

Balancing short-term economic needs with long-term climate goals remains a challenge. Recent shocks—such as the COVID-19 pandemic and regional instability—have diverted attention and resources away from climate policy. These disruptions exposed the fragility of existing strategies. Without stable and sustained investment, Croatia risks falling into reactive policymaking. Emergency responses may replace long-term planning.

## 5 Conclusions

This paper contributes to the limited research on the socio-economic impacts of climate policies in Croatia by assessing the country's current resilience to the climate crisis and the socio-economic constraints to climate-resilient development. It makes two key contributions to the literature: (i) it localizes the impact of climate change in Croatia and (ii) it explores the feedback between socio-economic development and climate change.

While existing studies emphasize the importance of investment in infrastructure, technology, and valuing natural capital, there has been limited focus on the availability of human capital necessary to implement these investments. This paper addresses the quantitative issue of capacity shortages in Croatia. Although the relevance of the skills gap is recognized, further research is needed to tackle this challenge comprehensively. Additionally, current adaptation policies assume that society will need to adapt to new circumstances but overlook structural changes in society, particularly in terms of its size and demographic composition. Migration (such as the influx of foreign workers) can help stabilize the size and age of the population, but entails challenges.

By linking sector-specific vulnerabilities—such as workforce shortages, productivity constraints, and exposure to climate extremes—with broader socio-economic trends, the paper demonstrates how these sectoral dynamics shape national capacity to design and implement effective climate policies. This connection is central to the overarching research objective: to assess how the feedback loop between climate change and socio-economic development constrains or enables climate policy outcomes.

This paper offers a methodological approach for identifying key sectors requiring structural economic reform, using criteria related to emissions reduction potential, climate vulnerability, and growth opportunities. It also provides an overview of sectoral vulnerabilities; however, regional vulnerabilities remain underexplored. Challenges in the Adriatic region, which heavily relies on tourism, differ from those in continental Croatia. Limited economic diversification within regions makes it more difficult for these areas to adapt to the climate crisis. Thus, analysis with a more granular geographic scope—focusing on regional differences in economic structure and climate—could further improve the results.

The Croatian case study shows tensions between economic development and ecological vulnerability, offering insights for Mediterranean and island nations (e.g., Greece, Thailand) that rely on climate-sensitive sectors like tourism. High-income yet low-resilience paradox mirrors challenges in Southern Europe, Australia, and California, while its EU membership highlights institutional misalignments between supranational climate agendas (e.g., the European Green Deal) and subnational demographic realities. These dynamics are relevant to aging societies (Japan, Germany) and post-industrial economies (Poland, Canada) navigating green transitions, emphasizing the need to integrate demographic strategies into climate governance.

## Data Availability

The original contributions presented in the study are included in the article/supplementary material, further inquiries can be directed to the corresponding authors.

## Footnotes

^1^*The National Development Strategy* (Official Gazette 13/2021) envisages faster GDP growth and reaching 75% of the EU average by 2030.

^2^Within 90 days of the entry into force of *the Electricity Market Act* (Official Gazette 111/2021), the Ministry of Economy and Sustainable Development received 216 applications for energy approval, which together include projects with a capacity of about 6 GW (Ministry of Economy and Sustainable Development, 2022a).

## References

[B1] Adom P. K. (2024). The Socioeconomic Impact of Climate Change in Developing Countries in the Next Decades: A Review. CGD Working Paper 681. Washington, DC: Center for Global Development. Available online at: https://www.cgdev.~org/publication/socioeconomic-impact-climate-change-developing-countries-next-decades-review (accessed January 15, 2025).

[B2] Asikainen T. Bitat A. Bol E. Czako V. Marimeier A. Muench S. Murasukaite-Bull I. Scapolo F.. (2021). The Future of Jobs is Green. Luxembourg: JRC.

[B3] Badanjak I. (2021). Workers Demand an End to Exploitation and an Increase in Labour Standards in Agriculture. Available online at: https://euractiv.jutarnji.hr/euractiv/hrana-i-poljoprivreda/radnici-traze-prestanak-eksploatacije-i-podizanje-standarda-rada-u-poljoprivredi-15068646 (accessed December 15, 2024).

[B4] Beslik S. Causevic A. (2019). Climate Risk Assessment Report: Croatia, A report commissioned by Nordea Group Sustainable Finance. Hrvatska gospodarska komora, Zagreb. Available online at: https://hgk.hr/documents/official-croatia-report5cef87495aaec.pdf (accessed January 3, 2025).

[B5] Bilal and Känzig. (2024). The Macroeconomic Impact of Climate Change: Global vs. Local Temperature. NEBR. Working Paper 32450. 10.3386/w3245034419315

[B6] Boehm L. Kläffling D. (2024). Social and Labour Market Impact of the Green Transition. Briefing. European Parliament. Available online at: https://www.europarl.europa.eu/RegData/etudes/BRIE/2024/762329/EPRS_BRI(2024)762329_EN.pdf (accessed January 15, 2025).

[B7] Boromisa A. M. (2022). “Energy governance in Croatia,” in Handbook of Energy Governance in Europe, eds. M. Knodt and J. Kemmerzell (Springer, Cham: New York). 10.1007/978-3-030-43250-8_5

[B8] BPIE (2018). Building 4 People: Quantifying the benefits of energy renovation investments in schools, offices and hospitals. Metodology and results. Available online at: https://bpie.eu/wp-content/uploads/2018/12/BPIE_methodology_031218.pdf (accessed January 15, 2025).

[B9] BPIE (2020). Building Renovation: a kick-starter for the EU recovery. Available online at: https://www.renovate-europe.eu/2020/06/10/building-renovation-a-kick-starter-for-the-eu-economy/ (accessed January 15, 2025).

[B10] Burke M. Hsiang S. M. Miguel E. (2015). Global non-linear effect of temperature on economic production. Nature 527, 235–239. 10.1038/nature15725 26503051

[B11] Carson M. Peterson G. eds. (2016). Arctic Resilience Report 2016. Stockholm Environment Institute and Stockholm Resilience Centre, Stockholm, Sweden, 218.

[B12] CEDEFOP (2021). The green employment and skills transformation—Insights from a European Green Deal skills forecast scenario, Publications Office of the European Union. Available online at: https://data.europa.eu/doi/10.2801/112540 (accessed January 15, 2025).

[B13] Chapagain P. S. Banskota T. R. Shrestha S. Khanal N. R. Yili Z. Yan J.. (2025). Studies on adaptive capacity to climate change: a synthesis of changing concepts, dimensions, and indicators. Humanit. Soc. Sci. Commun. 12:331. 10.1057/s41599-025-04453-320704490

[B14] Chen C. Noble I. Hellman J. Coffee J. Murillo M. Chawla N.. (2024a). University of *Notre Dame Global Adaptation Initiative. Country Index Technical Report*. ND-Gain. Available online at: https://gain.nd.edu/assets/581554/nd_gain_countryindex_technicalreport_2024.pdf (accessed January 15, 2025).

[B15] Chen K. de Schrijver E. Sivaraj S.. (2024b). Impact of population aging on future temperature-related mortality at different global warming levels. Nat. Commun. 15:1796. 10.1038/s41467-024-45901-z 38413648 PMC10899213

[B16] Croatian Forests (2022). Forests in Croatia. Available online at: https://www.hrsume.hr/sume/ (accessed January 15, 2025).

[B17] Croatian Parliament (2020). Climate Change Adaptation Strategy, Official Gazette, 46/2020. Zagreb.

[B18] Croatian Parliament (2022). Agriculture Strategy Until 2030 More than Just a Farm. NN 26/2022.

[B19] Dasgupta P. (2008). Nature's role in sustaining economic development. Environ. Resour. Econ. 39, 1–15. 10.1007/s10640-007-9178-4

[B20] Dasgupta P. (2021). The Economics of Biodiversity: The Dasgupta Review. London: HM Treasury.

[B21] Dell M. Jones B. F. Olken B. A. (2012). Temperature shocks and economic growth: evidence from the last half century. Am. Econ. J. Macroecon. 4, 66–95. 10.1257/mac.4.3.66

[B22] Deuster C. Kajander N. Muench S. Natale F. Nedee A. Scapolo F.. (2023). Demography and climate change, Joint Research Centre. Publications Office of the European Union, Luxembourg. Available online at: https://publications.jrc.ec.europa.eu/repository/handle/JRC133580 (accessed January 15, 2025).

[B23] Dietz S. Bowen A. Doda B. Gambhir J. Warren R. (2018). The economics of 1.5°C climate change. Annu. Rev. Environ. Resour. 43:2018. 10.1146/annurev-environ-102017-025817

[B24] DZS (2021a). Aktivno Stanovništvo U Republici Hrvatskoj U 2020. Prosjek Godine, Zagreb. Available online at: https://podaci.dzs.hr/2021/hr/10069 (accessed January 15, 2025).

[B25] DZS (2021b). Persons In Paid Employment, By Activities. December 2020. Broj/Number: 9.2.1/12. Available online at: https://web.dzs.hr/Hrv_Eng/publication/2020/09-02-01_12_2020.htm (accessed January 19, 2021).

[B26] DZS (2022a). Objavljeni Konačni Rezultati Popisa 2021. Available online at: https://dzs.gov.hr/vijesti/objavljeni-konacni-rezultati-popisa-2021/1270 (accessed January 15, 2025).

[B27] DZS (2022b). Active Population in the Republic of Croatia in 2021. Available online at: https://podaci.dzs.hr/2022/hr/29256 (accessed April 29, 2022).

[B28] DZS (2024). Aktivno Stanovništvo U Republici Hrvatskoj U 2023. Prosjek Godine, Zagreb. Available online at: https://podaci.dzs.hr/2024/hr/76780 (accessed January 15, 2025).

[B29] DZS (2025). Bruto Domaći Proizvod Za Republiku Hrvatsku, Hr_Nuts 2021. Hr Nuts 2 I Županije U 2022. Priopćenje. NR-2025-2-1, Veljača. Zagreb. Available online at: https://podaci.dzs.hr/2025/hr/97199 (accessed January 15, 2025).

[B30] EEA (2017). Report on the Estimated Climate Change Impacts and Vulnerability of Individual Sectors. EEAReport 1/2017. Available online at: https://www.eea.europa.eu/publications/climate-change-impacts-and-vulnerability-2016/at_download/file (accessed January 15, 2025).

[B31] EEA (2024). Economic losses and fatalities caused by weather - and climate - related extreme events (1980–2023)—per country. Available online at: https://www.eea.europa.eu/en/analysis/indicators/economic-losses-from-climate-related/economic-losses-and-fatalities-caused (accessed January 15, 2025).

[B32] EFIS Centre Technopolis Group and OldContinent (2024). European Innovation Scoreboard 2024 Country profile Croatia. European Commission, Brussels. Available online at https://ec.europa.eu/assets/rtd/eis/2024/ec_rtd_eis-country-profile-hr.pdf (accessed January 15, 2025).

[B33] Eurofound (2023). Fit for 55 Climate Package: Impact on EU Employment by 2030. Publications Office of the European Union, Luxembourg.

[B34] European Commission (2020). Commission Staff Working Document Impact Assessment Accompanying The Document Communication From The Commission To The European Parliament, The Council, The European Economic And Social Committee And The Committee Of The Regions Stepping up Europe's 2030 climate ambition Investing in a climate-neutral future for the benefit of our people. Document 52020SC0176, Brussels. SWD/2020/176 final. Available online at: https://eur-lex.europa.eu/legal-content/EN/TXT/?uri=CELEX:52020SC0176 (accessed January 15, 2025).

[B35] European Commission (2021a). Commission Notice- Technical guidance on preparing infrastructure for climate change in the period 2021-2027, 2021/C 373/01. Available online at https://eur-lex.europa.eu/legal-content/EN/TXT/HTML/?uri=CELEX:52021XC0916(03) (accessed January 15, 2025).

[B36] European Commission (2021b). Commission Delegated Regulation (EU) 2021/2139 of 4 June 2021 supplementing Regulation (EU) 2020/852 of the European Parliament and of the Council by establishing technical screening criteria to determine under which conditions an economic activity is considered to contribute substantially to climate change mitigation or adaptation and whether that economic activity causes significant harm to any other environmental objective (Text with EEA relevance), C/2021/2800, OJ L 442, 9.12.2021. Available online at: https://eur-lex.europa.eu/legal-content/HR/TXT/?uri=CELEX:32021R2139 (accessed January 15, 2025).

[B37] European Commission (2023a). Commission Recommendation of 18.12.2023 on the draft updated integrated national energy and climate plan of Croatia covering the period 2021-2030 and on the consistency of Croatia's measures with the Union's climate-neutrality objective and with ensuring progress on adaptation, {SWD(2023) 915 final}, C(2023) 9605 final, Brussels, 18.12.2023. Available online at: https://commission.europa.eu/document/download/6a6345e1-b20a-467d-ad13-185c6efaff8f_en?filename=Recommendation_draft_updated_NECP_Croatia_2023.pdf (accessed January 15, 2025).

[B38] European Commission (2023b). European Innovation Scoreboard 2023. Brussels: Publications Office of the European Union. Available online at: https://research-and-innovation.ec.europa.eu/statistics/performance-indicators/european-innovation-scoreboard_en (accessed January 15, 2025).

[B39] European Parliament and the Council (2018). Regulation (EU) 2018/1999 of the European Parliament and of the Council of 11 December 2018 on the Governance of the Energy Union and Climate Action.

[B40] Eurostat (2023a). Europop. Population projections update. Available online at: https://ec.europa.eu/eurostat/statistics-explained/index.php?title=Population_projections_in_the_EU#Population_projections_by_country (accessed January 15, 2025).

[B41] Eurostat (2023b). Gross Domestic Expenditure on RandD (GERD) as a Percentage of GDP. Luxembourg: Eurostat. Available online at: https://ec.europa.eu/eurostat/ (accessed January 15, 2025).

[B42] Eurostat (2024). Energy Imports Dependency. Available online at https://ec.europa.eu/eurostat/databrowser/view/nrg_ind_id/default/line?Lang=en (accessed January 15, 2025).

[B43] Eurostat (2025a). Real GDP per Capita. Online data code: sdg_08_10. Available online at: https://ec.europa.eu/eurostat/databrowser/view/sdg_08_10/default/table (accessed January 15, 2025).

[B44] Eurostat (2025b). Gross Domestic Product at Market Prices. Available online at: https://ec.europa.eu/eurostat/databrowser/view/tec00001/default/table?lang=en (accessed January 15, 2025).

[B45] Eurostat. (2022). Gross Domestic Product at Market Prices. https://ec.europa.eu/eurostat/databrowser/view/tec00001/default/table?lang=en (accessed May 15, 2023).

[B46] Fearden I. (1985). The Social Impact of Seasonal Employment in Devon and Cornwall. University of Plymuth. Available online at: https://pearl.plymouth.ac.uk/handle/10026.1/734 (accessed January 15, 2025).

[B47] Ferguson P. Wollersheim L. Lowe M. (2021). “Approaches to climate resilience,” in The Palgrave Handbook of Climate Resilient Societies, ed. R. C. Brears (Palgrave Macmillan, Cham). 10.1007/978-3-030-42462-6_97

[B48] Field C. B. Barros V. Stocker T. F. Dahe Q. eds. (2012). Managing the risks of extreme events and disasters to advance climate change adaptation: special report of the intergovernmental panel on climate change. Cambridge University Press. Available online at: https://www.ipcc.ch/report/managing-the-risks-of-extreme-events-and-disasters-to-advance-climate-change-adaptation/ (accessed January 15, 2025).

[B49] Gain.nd.edu. (2024). Country Index. Available online at: https://gain.nd.edu/our-work/country-index/rankings/ (accessed January 15, 2025).

[B50] Government of the Republic of Croatia (2020). Decision on the adoption of the Long-Term Strategy for the Renovation of the National Building Stock by 2050. NN 140/2020 (16.12.2020).

[B51] Government of the Republic of Croatia (2025). Odluku O Proglašenju Projekta ≫Prateća Infrastruktura Za Strateški Investicijski Projekt LNGg Terminal≪ Strateškim Investicijskim Projektom Republike Hrvatske, Zagreb NN 2/2025. Available online at: https://narodne-novine.nn.hr/clanci/sluzbeni/2025_01_2_9.html (accessed January 15, 2025).

[B52] Grizelj M. Akrap A. (2011). Projekcije stanovništva Republike Hrvatske od 2010. do 2061. Population projections of the Republic of Croatia 2010 – 2061. Croatian Bureau of Statistics, Zagreb.

[B53] Harlan S. L. Declet-Barreto J. H. Stefanov W. L. Petitti D. B. (2013). Neighborhood effects on heat deaths: Social and environmental predictors of vulnerability in Maricopa County, Arizona. Environ. Health Perspect. 121, 197–204. 10.1289/ehp.1104625 23164621 PMC3569676

[B54] Huggel C. Bouwer L. M. Juhola S. Mechler R. Muccione V. Orlove B. Wallimann-Helmer I. (2022). The existential risk space of climate change. Clim Change. 174:8. 10.1007/s10584-022-03430-y 36120097 PMC9464613

[B55] HZZ (2025). Statistika. Available online at: https://www.hzz.hr/statistika/ (accessed January 15, 2025).

[B56] IMF (2023). “Energy security and climate change: challenges and opportunities for Croatia,” in Epublic of Croatia: Selected Issues (Washington, DC). 10.5089/9798400246937.002

[B57] IPCC (2014). “Climate change 2014: mitigation of climate change.” in Contribution of Working Group III to the Fifth Assessment Report of the IPCC (Geneva: Intergovernmental Panel on Climate Change). 10.1017/CBO9781107415416

[B58] IPCC (2021). Climate change 2021. The Physical Science Basis. Contribution of Working Group I to the Sixth Assessment Report of the Intergovernmental Panel on Climate Change. Cambridge University Press. Available online at: https://www.ipcc.ch/report/ar6/wg1/ (accessed January 15, 2025).

[B59] IPCC (2022a). Climate Change 2022: Impacts, Adaptation, and Vulnerability. Contribution of Working Group II to the Sixth Assessment Report of the Intergovernmental Panel on Climate Change, eds. H.-O. Pörtner, D.C. Roberts, M. Tignor, E.S. Poloczanska, K. Mintenbeck, et al. (Cambridge University Press. Cambridge University Press, Cambridge, UK and New York, NY, USA), 3056.

[B60] IPCC (2022b). AR6 Climate Change 2022: Mitigation of Climate Change. Available online at: https://report.ipcc.ch/ar6/wg3/IPCC_AR6_WGIII_Full_Report.pdf (accessed January 15, 2025).

[B61] IRENA (2024). Croatia Country Profile. International Renewable Energy Agency. Abu Dhabi.

[B62] IRENA and ILO (2024). Renewable energy and jobs: Annual review 2024 International Renewable Energy Agency, Abu Dhabi, and International Labour Organization, Geneva. Available online at: https://www.irena.org/Publications/2024/Oct/Renewable-energy-and-jobs-Annual-review-2024 (accessed January 15, 2025).

[B63] Islam N. Winker J. (2017). Climate Change and Social Inequality. Department of Economic and Social Affairs. DESA Working Paper No. 152 United Nations32165543

[B64] JRC (2018). JRC Policy Insights, agricultural land abandonment in the EU within 2015–2030. Available online at: https://joint-research-centre.ec.europa.eu/system/files/2018-12/jrc113718.pdf (accessed January 15, 2025).

[B65] Kjellstrom T. Maître N. Saget C. Otto M. Karimova T. (2019). Working on a Warmer Planet: The Effect of Heat Stress on Productivity and Decent Work, ILO. Geneva. Available online at: https://www.ilo.org/publications/major-publications/working-warmer-planet-effect-heat-stress-productivity-and-decent-work (accessed January 15, 2025).

[B66] Marić J. (2021). Every fifth construction worker in Croatia is a foreigner. The number of Nepalese jumped sharply. Novi list. Availableonline at: https://www.novilist.hr/novosti/svaki-peti-radnik-zaposlen-na-gradevini-u-hrvatskoj-je-stranac-snazno-skocio-broj-nepalaca/ (accessed September 10, 2021).

[B67] Mastrorillo M. Licker R. Bohra-Mishra P. Fagiolo G. Estes L. D. Oppenheimer M. (2016). The influence of climate variability on internal migration flows in South Africa. Glob. Environ. Change 39, 155–169. 10.1016/j.gloenvcha.2016.04.014

[B68] Metcalf G. E. Weisbach D. (2009). The design of a carbon tax. Harv. L. Rev. 122, 2279–2331. 10.2139/ssrn.1324854

[B69] Ministry of Agriculture (2020). Annual Report on the State of Agriculture in 2019. Zagreb. Available online at: https://poljoprivreda.gov.hr/UserDocsImages/dokumenti/poljoprivredna_politika/zeleno_izvjesce/2020_11_30%20Zeleno%20izvje%C5%A1%C4%87e%202019.pdf (accessed January 15, 2025).

[B70] Ministry of Economy and Sustainable Development (2021). National Emission Inventory for 2021 Available online at: https://unfccc.int/documents/271575?gclid=CjwKCAiAmuKbBhA2EiwAxQnt732N6vHNpgKn7bmgamGsAk4DKpXlO1QaX8dQDxR_CAYPmom-z7ScKRoCLCAQAvD_BwE (accessed January 15, 2025).

[B71] Ministry of Economy and Sustainable Development (2022a). List of applications for the issuance of EOs. Available online at: https://mingor.gov.hr/UserDocsImages//UPRAVA%20ZA%20ENERGETIKU//Popis%20zahtjeva%20za%20izdavanje%20EO.pdf (accessed January 15, 2025).

[B72] Ministry of Economy and Sustainable Development (2022b). Draft assessments with recommendations to remove barriers and relieve administrative procedures that limit the increased use of renewable energy, June 2022. Available online at: https://esavjetovanja.gov.hr/ECon/MainScreen?entityId=20969 (accessed January 15, 2025).

[B73] Ministry of Economy and Sustainable Development (2023). National Inventory Report 2023, Croatian greenhouse gas inventory for the period 1990–2021. Available online at: https://unfccc.int/documents/627738 (accessed January 15, 2025).

[B74] Ministry of Physical Planning Construction and State Property. (2025). Database of Certified Installers of Renewable Energy Sources. Available online at: https://einstalaterioie.mpgi.hr/login.html (accessed January 15, 2025).

[B75] Ministry of Tourism and Sports (2023a). Sustainable Tourism Development Strategy until 2030. Official Gazzete 3/2023.

[B76] Ministry of Tourism and Sports (2023b). Turizam u brojkama. Available online at: https://mint.gov.hr/UserDocsImages/2023_dokumenti/230804_turizam_u_brojkama_2022_hrv.pdf (accessed January 15, 2025).

[B77] Munech S. Stermer E. Jensen K. Asikainen T. Salvi M. Scapolo F. (2022). Towards a green and digital future. Key requirements for successful twin transitions in the European Union. Publications Office of the European Union, Luexembourg, JRC129319

[B78] Murauskaite-Bull I. Scapolo F. Muench S. (2021). The Future of Jobs is Green. European Commission. Joint Research Centre. Publications Office.

[B79] Muttarak R. (2021). Demographic perspectives in research on global environmental change. Popul. Stud. 75, 77–10. 10.1080/00324728.2021.1988684 34902278

[B80] MuŽinić Marinić L. (2023). The impact of climate change on mental health. Soc. Psychiatry 51, 161–175.

[B81] Nordhaus W. D. (1992). An optimal transition path for controlling greenhouse gases. Science 258, 1315–1319. 10.1126/science.258.5086.1315 17778354

[B82] Office for Gender Equality of the Government of the Republic of Croatia (2022). National Plan for Gender Equality for the period from 2022 to 2027. Proposal. Available online at: https://esavjetovanja.gov.hr/Econ/MainScreen?EntityId=21788 (accessed January 15, 2025).

[B83] Pandey G. Lyden S. Franklin E. Harrison M. T. (2025). Agrivoltaics as an SDG enabler: trade-offs and co-benefits for food security, energy generation and emissions mitigation. Resour. Environ. Sustain 19:100186. 10.1016/j.resenv.2024.100186

[B84] Perpiña Castillo C. Kavalov B. Diogo V. Jacobs-Crisioni C. Batista e Silva F. Lavalle C. (2018). Territorial Facts and Trends in the EU Rural Areas within 2015-2030, Publications Office of the European Union, Luxembourg.

[B85] Plenković A. (2023). Although He Said that the Reconstruction is “Unrealistically Slow”, Plenković is not yet Sure Whether He Will Dismiss Paladina. Nacional. Available online at: https://www.nacional.hr/iako-je-rekao-da-je-obnova-nestvarno-spora-plenkovic-jos-nije-siguran-hoce-li-smijeniti-paladina/ (accessed August 1, 2023).

[B86] RCP database 2.0 (2025). Available online at https://tntcat.iiasa.ac.at/RcpDb/dsd?Action=htmlpageandpage=welcome (accessed January 15, 2025).

[B87] Riahi K. van V. D. P. Kriegler E. Edmonds J. O'Neill B. C. Fujimori S.. (2017). The Shared Socioeconomic Pathways and their energy, land use, and greenhouse gas emissions implications: an overview. Glob. Environ. Change 42, 153–168. 10.1016/j.gloenvcha.2016.05.009

[B88] Schwerdtle P. N. McMichael C. Mank I. Sauerborn R. Danquah I. Bowen K. J. (2020). Health and migration in the context of a changing climate: a systematic literature assessment. Environ. Res. Lett. 15. 10.1088/1748-9326/ab9ece33327439

[B89] Stabroek Y. Jansen H. (2021). Assessing the costs of climate change: a systematic review of the evidence. Environ. Res. Lett. 16:53001.

[B90] Stern N. H. (2006). The Economics of Climate Change: The Stern Review. London: Cambridge University Press. 10.1017/CBO9780511817434

[B91] Transparency International (2024). Corruption Perception Index. Available online at: https://www.transparency.org/en/cpi/2024(accessed January 15, 2025).

[B92] UN (2024). Data portal. Population division. World Population Prospects. Model based estimates 2021–2051. Available online at: https://population.un.org/dataportal/data/indicators/49/locations/191/start/2021/end/2051/table/pivotbylocation?df=f9bf736a-effd-4688-a4c4-fd73fe5c8e89 (accessed January 15, 2025).

[B93] UNDP (2020). Technical Report on SDG Finance Taxonomy. Available online at: https://www.undp.org/china/publications/technical-report-sdg-finance-taxonomy (accessed January 15, 2025).

[B94] UNEP (2021). Climate Change. Factsheet. Available online at: https://www.unep.org/unepmap/resources/factsheets/climate-change (accessed January 15, 2025).

[B95] UNHRC (2024). Analytical study on the impact of loss and damage from the adverse effects of climate change on the full enjoyment of human rights, exploring equity-based approaches and solutions to addressing the same. Report of the Secretary-General. United Nations Human Rights Council. Available online at: https://docs.un.org/en/A/HRC/57/30 (accessed January 15, 2025).

[B96] United Nations Office for Disaster Risk Reduction (2015). Sendai Framework for Disaster Risk Reduction 2015–2030. Cairo. Available online at: https://www.undrr.org/publication/sendai-framework-disaster-risk-reduction-2015-2030 (accessed January 15, 2025).

[B97] University of Notre Dame (2025). ND Gain, Country Rankings. Available online at: https://gain.nd.edu/our-work/country-index/rankings/ (accessed January 15, 2025).

[B98] Vandeplas A. Vanyolos I. Vigani M. Vogel L. (2022). The Possible Implications of the Green Transition for the EU Labour Market, European Commission.

[B99] Wachsmuth J. Alexander-Haw A. Billerbeck A. Breitschopf B. Brunzema I. Berger C.. (2023). National Energy and Climate Plans: Evidence of Policy Impacts and Options for more Transparency. A Meta Study assessing Evaluations of selected Policies reported in the Danish, French, German, Slovenian, and Swedish Plan. Final report. Dessau-Roßlau: German Environment Agency. Available online at: https://www.ecologic.eu/19479 (accessed January 15, 2025).

[B100] Walker B. Carpenter S. Anderies J. Abel N. Cumming G. Janssen M.. (2002). Resilience management in social-ecological systems: a working hypothesis for a participatory approach. Conserv. Ecol. 6:14. 10.5751/ES-00356-06011430174746

[B101] WMO (2022). Reported extreme events in 2021. Available online at: https://wmo.maps.arcgis.com/apps/instant/interactivelegend/index.html?appid=43b371aeb7af4c8b9e9c77a4370a13ed (accessed January 15, 2025).

[B102] World Bank (2022). Croatia- Impact sea level rise. Available online at: https://climateknowledgeportal.worldbank.org/country/croatia/impacts-sea-level-rise (accessed January 15, 2025).

[B103] World Bank (2024a). Climate knowledge portal. Climate data projections. Available online at: https://climateknowledgeportal.worldbank.org/country/croatia/climate-data-projections (accessed January 15, 2025).

[B104] World Bank (2024b). Data from database: Population estimates and projections. Available online at: https://databank.worldbank.org/source/population-estimates-and-projections# (accessed December 16, 2024).

[B105] World Economic Forum (2021). The Global Risks Report 2021. (15th ed). World Economic Forum.

[B106] World Economic Forum (2023). The Future of Jobs Report. World Economic Forum.

